# A Hierarchical Model of Inhibitory Control

**DOI:** 10.3389/fpsyg.2018.01339

**Published:** 2018-08-02

**Authors:** Jeggan Tiego, Renee Testa, Mark A. Bellgrove, Christos Pantelis, Sarah Whittle

**Affiliations:** ^1^Monash Institute of Cognitive and Clinical Neurosciences, School of Psychological Sciences, Monash University, Clayton, VIC, Australia; ^2^Melbourne Neuropsychiatry Centre, Department of Psychiatry, University of Melbourne & Melbourne Health, Carlton South, VIC, Australia; ^3^Florey Institute for Neuroscience and Mental Health, Parkville, VIC, Australia; ^4^Centre for Neural Engineering, Department of Electrical and Electronic Engineering, University of Melbourne, Parkville, VIC, Australia; ^5^Melbourne School of Psychological Sciences, The University of Melbourne, Parkville, VIC, Australia

**Keywords:** inhibitory control, inhibition, response inhibition, attentional inhibition, working memory capacity

## Abstract

Inhibitory control describes the suppression of goal-irrelevant stimuli and behavioral responses. Current developmental taxonomies distinguish between Response Inhibition – the ability to suppress a prepotent motor response, and Attentional Inhibition – the ability to resist interference from distracting stimuli. Response Inhibition and Attentional Inhibition have exhibited moderately strong positive correlations in previous studies, suggesting they are closely related cognitive abilities. These results may reflect the use of cognitive tasks combining Stimulus–Stimulus- and Stimulus–Response-conflict as indicators of both constructs, which may have conflated their empirical association. Additionally, previous statistical modeling studies have not controlled for individual differences in Working Memory Capacity, which may account for some of the empirical overlap between Response Inhibition and Attentional Inhibition. The aim of the current study was to test a hierarchical model of inhibitory control that specifies Working Memory Capacity as a higher-order cognitive construct. Response Inhibition and Attentional Inhibition were conceptualized as lower-order cognitive mechanisms that should be empirically independent constructs apart from their shared reliance on Working Memory Capacity for active maintenance of goal-relevant representations. Measures of performance on modified stimulus–response compatibility tasks, complex memory span, and non-selective stopping tasks were obtained from 136 preadolescent children (*M* = 11 years, 10 months, *SD* = 8 months). Consistent with hypotheses, results from Structural Equation Modeling demonstrated that the Response Inhibition and Attentional Inhibition factors were empirically independent constructs that exhibited partial statistical dependence on the Working Memory Capacity factor. These findings have important implications for current theories and models of inhibitory control during development.

## Introduction

In developmental cognitive psychology, ‘inhibitory control’ is an umbrella term used to describe the voluntary control, or *inhibition*, of goal-irrelevant stimuli, cognitions, and behavioral responses (Nigg, 2000; Diamond, 2013). It is a construct central to several theoretical accounts of cognitive development and developmental psychopathology (Bjorklund and Harnishfeger, 1990; Schachar and Logan, 1990; Dempster, 1992; Pennington and Ozonoff, 1996; Nigg, 2000, 2017). However, the concept of inhibitory control is often over-extended to encompass a broad range of distinct cognitive processes, potentially limiting its utility as an explanatory construct (Nigg, 2000; Aron, 2007). The term has also been applied to cognitive processes, such as selectively attending to competing visual stimuli or representations in working memory, in which direct inhibition is unlikely to be the underlying neurobiological mechanism (Aron, 2007; Munakata et al., 2011). Previous attempts have been made to provide conceptual clarity and introduce a theoretical framework for understanding inhibitory control in developmental and adult samples (Nigg, 2000; Friedman and Miyake, 2004). However, several key issues remain unaddressed, such as the inter-relationships and structural organization between putatively distinct inhibitory control processes, as well as their associations with working memory (Diamond, 2013). Clarity on these issues may assist in advancing theory in developmental cognitive psychology and related fields.

### Response Inhibition and Attentional Inhibition

Two key cognitive processes that have been investigated under the rubric of inhibitory control are: (1) Response Inhibition [also referred to as ‘Behavioral Inhibition,’ ‘Motor Inhibition,’ ‘Prepotent Response Inhibition,’ and ‘(Attention) Restraint’]; and (2) Attentional Inhibition [also referred to as ‘Interference Control,’ ‘Interference Suppression,’ ‘Resistance to (Distracter) Interference,’ and ‘Attention Constraint’] (Nigg, 2000; Friedman and Miyake, 2004; Wiebe et al., 2008; Gandolfi et al., 2014; Stahl et al., 2014; Kane et al., 2016). See **Table 1** for different terminology used to refer to the same cognitive processes across different theoretical approaches and studies (Friedman and Miyake, 2004; Diamond, 2013; Nigg, 2017). Response Inhibition refers to the process of countermanding a prepotent motor response and has generally been assessed using non-selective stopping tasks, such as the stop signal, go/no-go, and antisaccade tasks, which require participants to intermittently suppress a motor response given presentation of a conditional stimulus or cue (Verbruggen and Logan, 2008; Chambers et al., 2009; Aron et al., 2014). Attentional Inhibition refers to the ability to resist interference from stimuli in the external environment, and has been investigated using visual matching tasks that require participants to judge whether target and comparison stimuli are the same or different whilst ignoring task-irrelevant distracters (Friedman and Miyake, 2004; Stahl et al., 2014; Nigg, 2017).

**Table 1:** Comparison of terms used across studies to describe the two inhibitory control constructs of interest.

Present study	Nigg (2000)	Friedman and Miyake (2004)	Diamond (2013)	Gandolfi et al. (2014)	Stahl et al. (2014)	Kane et al. (2016)	Nigg (2017)
Response Inhibition	Behavioral Inhibition	Prepotent Response Inhibition	Behavioral Inhibition/Behavioral Self-Control	Response Inhibition	Behavioral Inhibition	Attention Restraint	Response Inhibition
Attentional Inhibition	Interference Control	Resistance to Distracter Interference	Inhibition of Attention/Selective, Focused Attention	Interference Suppression	Stimulus Interference	Attention Constraint	Attentional Inhibition

Response Inhibition and Attentional Inhibition are also commonly measured using performance on Stimulus–Response Compatibility (SRC) tasks, such as the Stroop, Eriksen Flanker (Flanker), and Simon tasks (Stroop, 1935; Simon, 1969; Eriksen and Eriksen, 1974). These are forced-choice reaction time tasks that require participants to selectively attend and respond to target stimuli whilst ignoring goal-irrelevant distracting stimuli and response options on interspersed trials. SRC tasks generally consist of three main conditions: (1) control; (2) congruent (or compatible); and (3) incongruent (or incompatible), which are defined according to the correspondence or non-correspondence between task-relevant and task–irrelevant stimulus and response elements (Kornblum et al., 1990; MacLeod, 1991; Eriksen, 1995; Lu and Proctor, 1995). Control trials are characterized by presentation of task-relevant stimuli, or stimulus dimensions, in the absence of task-irrelevant stimuli and response features. In contrast, task-irrelevant stimuli and response alternatives appear in congruent and incongruent trials. On congruent trials task-relevant and task-irrelevant stimuli and response features correspond, such that stimulus identification and response generation can occur automatically (Kornblum, 1994; Kornblum and Lee, 1995). In contrast, on incongruent trials there is a mismatch between the task-relevant and task-irrelevant stimuli and response tendencies, which compete for further processing. Mean reaction times on incongruent trials are typically longer than on congruent or control trials (Kornblum et al., 1990; MacLeod, 1991; Eriksen, 1995; Lu and Proctor, 1995). The difference in mean reaction time is called the ‘*interference effect’* and reflects the additional time required to resolve the conflict between competing stimulus representations and response tendencies (Fan et al., 2002; Rueda et al., 2004). Studies have used the interference effect obtained from performance of the Stroop and Simon tasks to measure Response Inhibition and variants of the Flanker task to measure Attentional Inhibition (Friedman and Miyake, 2004; Forstmann et al., 2008; Kane et al., 2016).

### Previous Studies of Response Inhibition and Attentional Inhibition

Several studies have attempted to clarify the distinction and empirical relationship between Response Inhibition and Attentional Inhibition. In their seminal and highly cited study, Friedman and Miyake (2004) used Structural Equation Modeling (SEM) to investigate the relationship between these two constructs, which they called ‘Prepotent Response Inhibition’ and ‘Resistance to Distractor Interference,’ as well as a third putative inhibition-related function ‘Resistance to Proactive Interference’ – the ability to resolve interference from previously encountered information. Performance data was obtained from 220 undergraduate students aged 18 – 40 years across nine cognitive tasks proposed as measuring the three constructs of interest. Response Inhibition was measured using the stop-signal, antisaccade, and Stroop tasks, and Resistance to Distracter Interference was measured using two stimulus matching tasks and the Flanker task. Results demonstrated that Prepotent Response Inhibition and Resistance to Distracter Interference were closely associated (*r* = 0.67) and could be collapsed into a unitary construct called ‘Response-Distracter Inhibition.’ Friedman and Miyake (2004, p. 125) contended that these results provided evidence for a “*common inhibition ability*,” reflecting a shared reliance on active goal maintenance or Working Memory Capacity (WMC). This theoretical perspective suggests that goal-relevant information is actively maintained in a readily accessible state in working memory through mechanisms of executive attention (Kane and Engle, 2002). The capacity to actively maintain goal-related information is seen to be a fundamental aspect of top-down control, because it purportedly enables individuals to override automatic stimulus selection and response execution and engage in goal-directed cognition and behavior (Kane et al., 2001; Engle, 2002; Kane and Engle, 2002; Kane et al., 2007). The work of Friedman and Miyake (2004) therefore attributed the ability to selectively attend to task-relevant stimuli and inhibit task-irrelevant responses to the same common and unidimensional goal maintenance factor. However, because this study did not include measures of WMC, the proposed common reliance of Response Inhibition and Attentional Inhibition on active goal maintenance could not be explicitly tested.

A study conducted by Kane et al. (2016) using SEM provided a theoretical and methodological extension to the Friedman and Miyake (2004) model. This study included two constructs analogous to the Prepotent Response Inhibition and Resistance to Distracter Interference factors of Friedman and Miyake (2004), which the authors called ‘Attention Restraint’ and ‘Attention Constraint,’ respectively (Kane et al., 2016). Measures of the Attention Restraint factor included a combination of antisaccade and Stroop task variants, and several Flanker task variables were used to measure the Attention Constraint factor. In addition, the Kane et al. (2016) study included six complex memory span tasks, which consist of the presentation of to-be-remembered items interleaved with a secondary processing task. Complex memory span tasks enable measurement of individual differences in the executive attention component of working memory, which is engaged to maintain and protect the memory items against temporal decay and interference from the secondary processing task (Conway et al., 2005). Inclusion of these tasks in the Kane et al. (2016) study allowed the association between Attention Constraint and Attention Restraint to be estimated whilst also examining their relationship with WMC. The results demonstrated significant associations between the WMC, Attention Constraint (*r* = -0.64) and Attention Restraint (*r* = -0.40) factors and revealed a moderately strong positive correlation between these latter two constructs (*r* = 0.60), comparable in strength to that obtained by Friedman and Miyake (2004). Kane et al. (2016) found that constraining the correlation between Attention Constraint and Attention Restraint to one resulted in non-convergence of the model, indicating that these two factors represented distinguishable, but empirically related, constructs. However, these researchers did not regress the Attention Constraint and Attention Restraint factors onto WMC; thus, it is unclear whether their correlation was partly or fully explained by a shared statistical dependence on the WMC factor.

Extending examination of inhibitory control to a developmental sample using age-appropriate cognitive tasks, Gandolfi et al. (2014) successfully differentiated these two constructs, which they called Response Inhibition and Interference Suppression, in toddlers aged 36 – 48 months. However, these two constructs were also moderately strongly correlated (*r* = 0.71), indicating substantial empirical overlap. Furthermore, without inclusion of tasks measuring WMC, it is unclear whether this intercorrelation was attributable to a shared empirical overlap with WMC. Response Inhibition and Interference Suppression were also not yet differentiated in toddlers aged 24 – 32 months, suggesting increased functional segregation of component inhibitory control processes with ongoing development, similar to the divergent developmental trajectory and increased modularity observed for other executive functions (Best and Miller, 2010; Lee et al., 2013). There have been no other studies specifically examining the relationship between Response Inhibition and Attentional Inhibition in a developmental sample.

### Limitations of Previous Studies

A limitation of these previous studies examining the interrelationship between inhibitory control constructs is a failure to account for the task impurity problem, in which multiple cognitive processes are engaged during performance of complex cognitive tasks (Friedman and Miyake, 2004). The Dimensional Overlap taxonomy (Kornblum et al., 1990) specifies two distinct forms of attentional conflict arising during performance of SRC tasks commonly used to measure Response Inhibition and Attentional Inhibition: *Stimulus–Stimulus-* (‘*SS-*’) *conflict* and *Stimulus–Response-* (‘*SR-*’) *conflict*. SS-conflict reflects competition between the relevant and irrelevant stimuli or stimulus dimensions (i.e., Attentional Inhibition), whereas SR-conflict reflects competition between the correct, task-relevant response and the prepotent incorrect response (i.e., Response Inhibition) (Kornblum et al., 1990; Zhang et al., 1999). The studies conducted by Friedman and Miyake (2004), Gandolfi et al. (2014), and Kane et al. (2016) have used SRC tasks combining SS-conflict and SR-conflict as measures of both Response Inhibition and Attentional Inhibition, which may have conflated the empirical relationship between these two constructs. For example, variants of the Flanker task were used in all three studies as a measure of Attentional Inhibition (Friedman and Miyake, 2004; Kane et al., 2016). However, interference effects arising during performance on incongruent trials of the Flanker task have been attributed predominantly to SR-conflict (Eriksen and Eriksen, 1974; Eriksen and St James, 1986; Eriksen, 1995; van Veen et al., 2001). Conversely, the Stroop task was used as a measure of Response Inhibition by Friedman and Miyake (2004) and Kane et al. (2016). However, a large proportion of the interference effect on standard Stroop task trials is attributable to SS-conflict (Simon and Berbaum, 1990; Zhang et al., 1999; Milham et al., 2001; Egner et al., 2007). The use of standard interference effects on the Flanker and Stroop task as indicators of the Response Inhibition and Attentional Inhibition factors, may have conflated their empirical association and rendered these constructs more difficult to dissociate empirically.

A study conducted by Stahl et al. (2014) addressed this limitation by using three variants of a visual matching task that consisted of only SS-conflict as measures of the Attentional Inhibition construct, which they called ‘Stimulus Interference.’ The results of SEM conducted in a sample of 198 adults aged 18 – 48 years demonstrated that this Stimulus Interference factor was not significantly correlated with a Response Inhibition factor, which they called ‘Behavioral Inhibition,’ measured using the stop-signal, go/no-go, and antisaccade tasks (Stahl et al., 2014). Attentional Inhibition can also be measured with greater specificity by using modified SRC tasks that enable SS-conflict to be differentiated and examined separately from SR-conflict (Milham et al., 2001; van Veen et al., 2001; De Houwer, 2003). Separate measurement of SS-conflict as a more reliable and specific measure of Attentional Inhibition may enable the empirical independence of this construct from Response Inhibition to be demonstrated in a developmental sample.

Another limitation of previous studies is that they have not statistically controlled for the common empirical association of Response Inhibition and Attentional Inhibition with WMC (Friedman and Miyake, 2004; Gandolfi et al., 2014; Kane et al., 2016). From this perspective, the Response Inhibition and Attentional Inhibition factors would be regressed onto the WMC factor in a hierarchical structural regression model, reflecting their shared statistical dependence on individual differences in executive attention and active goal-maintenance (Kane et al., 2001; Engle, 2002). WMC would then be represented as a higher-order factor that supports Response Inhibition and Attentional Inhibition as distinct lower-order constructs. The Response Inhibition and Attentional Inhibition factors would be predicted to be empirically independent, apart from this shared dependence on WMC. This proposed hierarchical model of inhibitory control, with WMC specified as a higher-order construct, would concord with previous accounts in which the executive component of working memory (i.e., the ‘Central Executive’) represents a domain-general attentional resource that supports lower-order, domain-specific cognitive processes (Baddeley, 2002, 2010; Kane and Engle, 2002; Repovs and Baddeley, 2006). The hierarchical model is also supported empirically by observations that efficiency of visual search and selective attention, as well as the relative success of inhibiting prepotent responses, are sensitive to manipulations of attentional load in working memory, as well as being linked to individual differences in WMC (Roberts et al., 1994; Kane et al., 2001; Unsworth et al., 2004; Lavie, 2005; Burnham et al., 2014).

### Developmental Changes in Inhibitory Control

Due to a paucity of research, it is currently unclear how developmental changes affect the relationship between Response Inhibition and Attentional Inhibition. Response Inhibition exhibits a protracted developmental trajectory, with rapid maturation in early childhood followed by gradual improvements throughout adolescence that continue into early adulthood (Williams et al., 1999; Best and Miller, 2010; Luna et al., 2015). Substantial increases in WMC, as measured by complex memory span tasks, are observed between the ages of 5 and 11 years, with smaller but significant increases from age 11 until age 15 when adult levels of performance are typically reached (Gathercole et al., 2004; Gathercole and Alloway, 2008). Attentional Inhibition may improve in concert with developmental increases in WMC, however, very little research has been conducted in this area (Stedron et al., 2005; Diamond, 2013). The childhood to adolescent transition may be a critical period in which to study the interrelationships of Response Inhibition and Attentional Inhibition given the slower developmental increases in WMC, and possibly Attentional Inhibition, during this time (i.e., after 11 years of age).

### The Current Study

The aim of the current study was to test a model of inhibitory control in a developmental sample that differentiates between Response Inhibition and Attentional Inhibition as empirically independent constructs that are hierarchically organized, reflecting a shared dependence on WMC. Based on the theoretical and empirical framework introduced above it was expected that both the Response Inhibition and Attentional Inhibition factors would be significantly predicted by the WMC factor in a structural regression model, reflecting their shared dependence on active goal maintenance. It was also hypothesized that Response Inhibition and Attentional Inhibition would be empirically independent constructs after being regressed onto WMC, after which their shared variance with this factor would be accounted for. These hypotheses were tested in a pre-adolescent sample of children aged 11 and 12 years, given (a) the lack of prior research in young samples; (b) the importance of inhibitory control to cognitive development and developmental psychopathology; (c) the relative plateau in development of WMC after age 11 years. While the development of inhibitory control is a topic of interest, a restricted age range was employed here as a first step toward better understanding these constructs in children.

## Materials and Methods

### Participants

The sample consisted of 136 (125 right-handed) typically developing, pre-adolescent children (86 males, 50 females), aged 11 years, 0 months to 12 years, 11 months (*M* = 11 years, 10 months, *SD* = 8 months) that were tested as part of a larger study on cognition, self-regulation, and emotional and behavioral problems. Parents and caregivers nominated themselves and their child for participation in the study based on their child meeting the eligibility criteria requiring that they be aged between 11 years 0 months and 12 years 11 months; have normal, or corrected to normal, vision and hearing; speak English as his/her first language; have no history of a diagnosed learning disorder, brain injury, or psychiatric illness; and not currently be taking any psychoactive medications. Recruitment took place between December 2013 and April 2015 across year levels 5 (32.6%), 6 (37.9%), and 7 (29.5%) from 52 primary and secondary schools located in metropolitan Melbourne, Australia using advertisement flyers handed out to students in class and through school newsletters. Student participants largely attended Government schools (63.8%), with a smaller number recruited from Catholic (30.8%), and Independent (5.4%) schools. The majority (86%) of the student sample were of European descent. Sample size selection was based on a recommended minimum case-to-parameter ratio (*N*:*q*) of 10:1, required for obtaining parameter estimates with reasonable precision (Jackson, 2003; Kline, 2016). This study was carried out in accordance with the Australian Code for the Responsible Conduct of Research and the National Statement on Ethical Conduct in Human Research as outlined by the National Health and Medical Research Council. Ethics approval for the research project and associated methodology was obtained from the Monash University Human Research Ethics Committee (Approval Number: CF13/1307 - 2013000673), the Department of Education and Early Childhood Development (Approval Number: 2013_002137), and the Catholic Education Office, Archdiocese of Melbourne (Approval Number: GE13/0009, Project# 1947). Consent to approach individual schools was granted by Independent Schools of Victoria (July 5th, 2013). Written informed consent to conduct the research was also obtained from the principals of participating schools. All research participants gave written informed consent in accordance with the Declaration of Helsinki. Informed written assent to participate was obtained from student participants, and written informed consent was also obtained from their parents, or legal guardians.

### Materials

#### Automated Working Memory Assessment (AWMA) – Verbal Working Memory Subtests

The Automated Working Memory Assessment (AWMA) is a computerized test battery for assessing WMC in young persons aged 4–22 years in both verbal and visuospatial domains (Alloway, 2007). The AWMA was administered on a Dell Inspiron 1520 computer with 33 cm screen at 1280 × 800 screen using the Windows Vista operating system and operated by one of the investigators. The verbal stimuli for the subtests were presented at a fixed volume for each participant. WMC was assessed using two complex verbal memory span tests, Listening Recall, and Counting Recall, and a transformation span task, Backward Digit Recall, which all have demonstrated reliability and validity as measures of verbal working memory in child and adolescent samples (Gathercole et al., 2004; Alloway et al., 2006). The Listening Recall subtest is an auditory version of the Reading Span task originally developed by Daneman and Carpenter (1980). Participants are required to recall, in correct order, a list of orally presented words that are interleaved with a secondary processing task, consisting of true/false judgments regarding whether presented sentences make sense. Counting Recall, based on the task created by Case et al. (1982), required participants to count the number of red-colored circles and ignore the blue triangles presented on each trial and then recall each of these numbers in correct order following a delay. Backward Digit Recall required participants to repeat a sequence of orally presented digits of varying length in reverse order. Set sizes varied from two to eight and participants were required to respond correctly to four of six trials at each level of difficulty to advance to the next set. The dependent variables were the subtest raw scores reflecting the number of correctly answered trials. Participants were given clear instructions and an opportunity to practice each task before commencing.

#### Response Inhibition Tasks

##### Stop signal task

The stop signal task was administered using the Stop-It and Analyze-It program (Verbruggen et al., 2008) installed on a Dell Latitude D420 Laptop computer running Windows XP. Each participant was provided with standardized instructions prior to testing designed to minimize strategic responding (Logan et al., 1997; Williams et al., 1999). A practice block of 32 trials was followed by 3 experimental blocks consisting of 64 trials each. There was a 10 s interval between blocks during which participants were given feedback on their performance on previous blocks in terms of percentage correct responses and mean reaction time. This feedback was designed to encourage participants to maintain speed and accuracy in their pattern of responding throughout the task. Each trial began with the display of a white fixation cross in the center of the blank screen for a period of 250 milliseconds (ms). The fixation cross was followed by presentation of a visual stimulus in the form of either a white square or circle, which was displayed on a black background until participants responded or until the maximum reaction time of 1250 ms had elapsed. On 75% of the trials, called no-signal trials, participants were required to indicate as quickly and as accurately as possible whether a square or circle had been presented with a left- (“*z*” key) or right-handed (“*/*” key) keyboard response, respectively. Following a response, or after the maximum reaction time had elapsed, there was an interval of 2000 ms prior to presentation of the subsequent trial stimulus.

On 25% of the trials, called stop-signal trials, a 750 Hz auditory stop-signal of 75 ms duration followed stimulus presentation at varying delays indicating that participants should inhibit their response to the task on that trial. The varying interval for presentation of the stop-signal is called the Stop-Signal Delay (SSD) and represents a handicap for the inhibitory process in order to reduce the probability of successful inhibition (Logan and Cowan, 1984; Logan et al., 1997). SSD is dynamically adjusted using an inbuilt staircase tracking procedure to converge on a delay at which the overall probability of successful inhibition on the stop-signal task is approximately 50% (Verbruggen et al., 2008). Stop Signal Reaction Time (SSRT) represents a measure of the efficiency of the inhibitory process, with faster times reflecting greater inhibitory control (Verbruggen and Logan, 2009). Stop-It uses the subtraction method for calculating SSRT, in which mean SSD is subtracted from mean reaction time on no-signal trials (Logan et al., 1997; Verbruggen et al., 2008). Following performance of the stop-signal task Analyze-It was used to ensure that for each participant the probability of inhibition was not significantly different from 0.5 (*Z*< ± 1.96, *p* > 0.05), such that the subtraction method could be used to accurately calculate SSRT as the dependent variable (Verbruggen et al., 2008).

##### Go/no-go task

The go/no-go task used in the current study was adapted from the simple go/no-go task previously used by Menon et al. (2001) and described in Chambers et al. (2009). It was programmed using PsychoPy V1.80.03 (Peirce, 2007, 2008) and administered on the same computer as the AWMA. The task consisted of two ‘go’ stimuli, the letters ‘Y’ and ‘Z,’ and one ‘no-go’ stimulus, the letter ‘X’ all presented in white. The height of these stimuli subtended 5° of visual angle. Each trial began with presentation of the stimulus at central fixation for 750 ms, followed by a 1000 ms inter-stimulus interval consisting of a blank screen, which preceded the subsequent trial. Trials on which a ‘Y’ or ‘Z’ appeared required participants to respond as quickly as possible by pressing the ‘*H*’ key on the computer keyboard. Trials on which participants failed to register their response during the interval of stimulus presentation were registered as omission errors. Conversely, participants were required to inhibit their key press on trials where an ‘X’ appeared. Responses on no-go trials were scored as commission errors. There were 5 practice trials followed by two blocks of 100 trials each, which consisted of 75% go and 25% no-go trials. Trials were also ordered such that there were no more than two consecutive no-go trials. The combination of a large number and high ratio of interspersed go to no-go trials, as well as minimal consecutive no-go trials, assisted in building up a strong prepotent response requiring the engagement of inhibitory processes (Simmonds et al., 2008). The dependent variable was the number of commission errors on no-go trials.

##### Simon task

**FIGURE 1:**
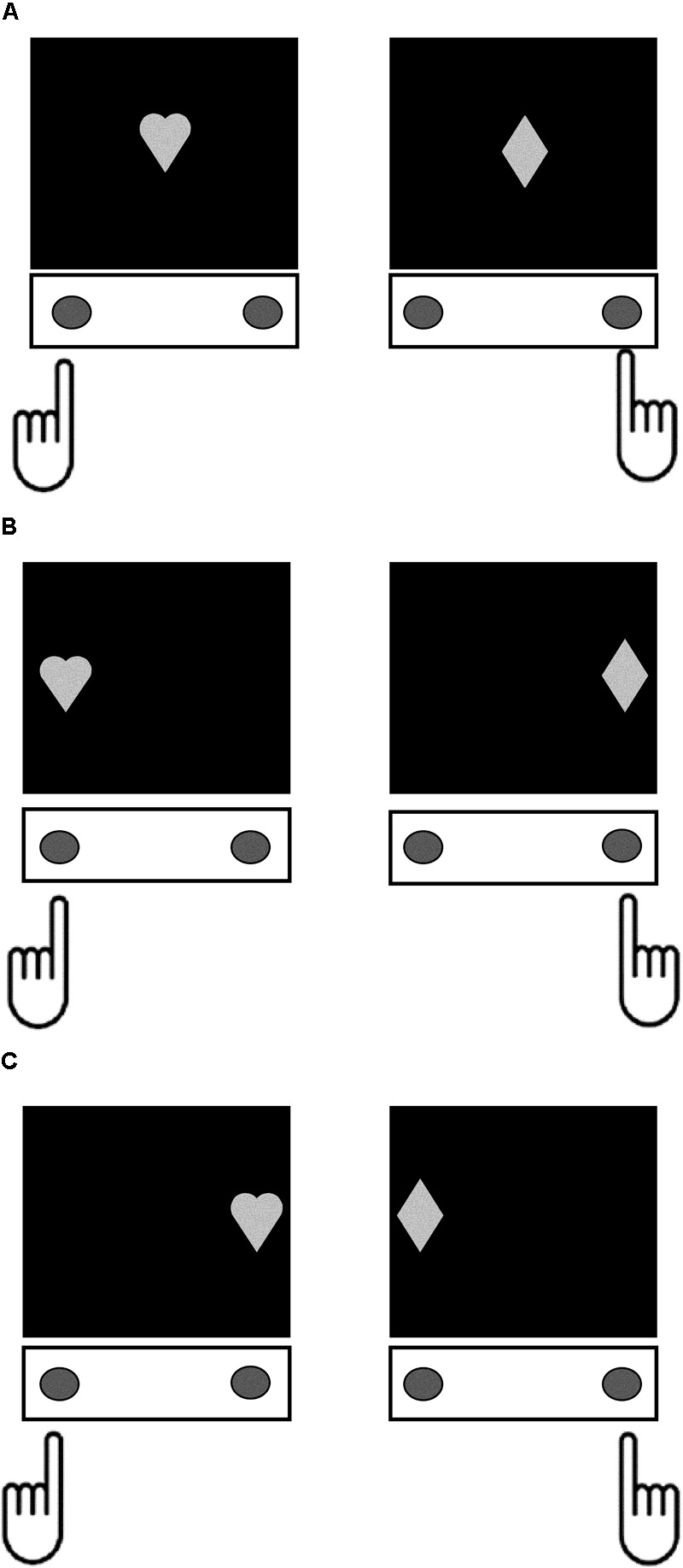
Depiction the different trial conditions in the Simon task. For each trial type, participants responded by pressing the button under their left index finger when the stimulus was a Heart, and the button under their right index finger when the stimulus was a Diamond. **(A)** Control condition – the target stimuli appeared at central fixation. **(B)** Congruent condition – the target stimuli appeared at a location on the screen that was compatible with the spatial orientation of the correct response (left side for Heart and right side for Diamond). **(C)** Incongruent condition – the target stimuli appeared at a location on the screen that was incompatible with the spatial orientation of the correct response (right side for Heart and left side for Diamond).

The Simon task (Sparkes, 2006) was also programmed using PsychoPy V1.80.03 (Peirce, 2007, 2008) and administered on the same computer as the go/no-go task. Stimuli were a white-colored heart and diamond, with height and width subtending 7.5° of visual angle, and mapped onto left and right-handed responses, respectively. These stimuli could be presented at central fixation in the control condition, or 10° to the left or right of central fixation in the congruent and incongruent conditions (**Figure 1**). In the congruent condition, participants were presented with a stimulus in a spatial location that was compatible with the correctly mapped response (Heart on the left, or Diamond on the right). In the incongruent condition, participants were presented with a stimulus in a spatial location that was incompatible with the correctly mapped response (Heart to the right, or Diamond to the left). There were 6 practice trials followed by two experimental blocks containing 20 control, 60 congruent, and 60 incongruent trials in total. Each trial began with a fixation cross for 500 ms, followed by presentation of the stimulus until response, and a 500 ms inter-stimulus interval. The dependent variable was the difference in mean reaction times on incongruent compared to control trials (Simon SR-conflict). Behavioral selective stopping on the Simon task engages overlapping neurocognitive mechanisms to non-selective stopping on the stop-signal and go/no-go tasks (Aron and Verbruggen, 2008; Forstmann et al., 2008). Thus, SR-conflict on the Simon task is proposed to provide a measure of the same Response Inhibition construct as the stop-signal and go/no-go tasks.

#### Attentional Inhibition Tasks

##### Modified Stroop color-word interference test

**FIGURE 2:**
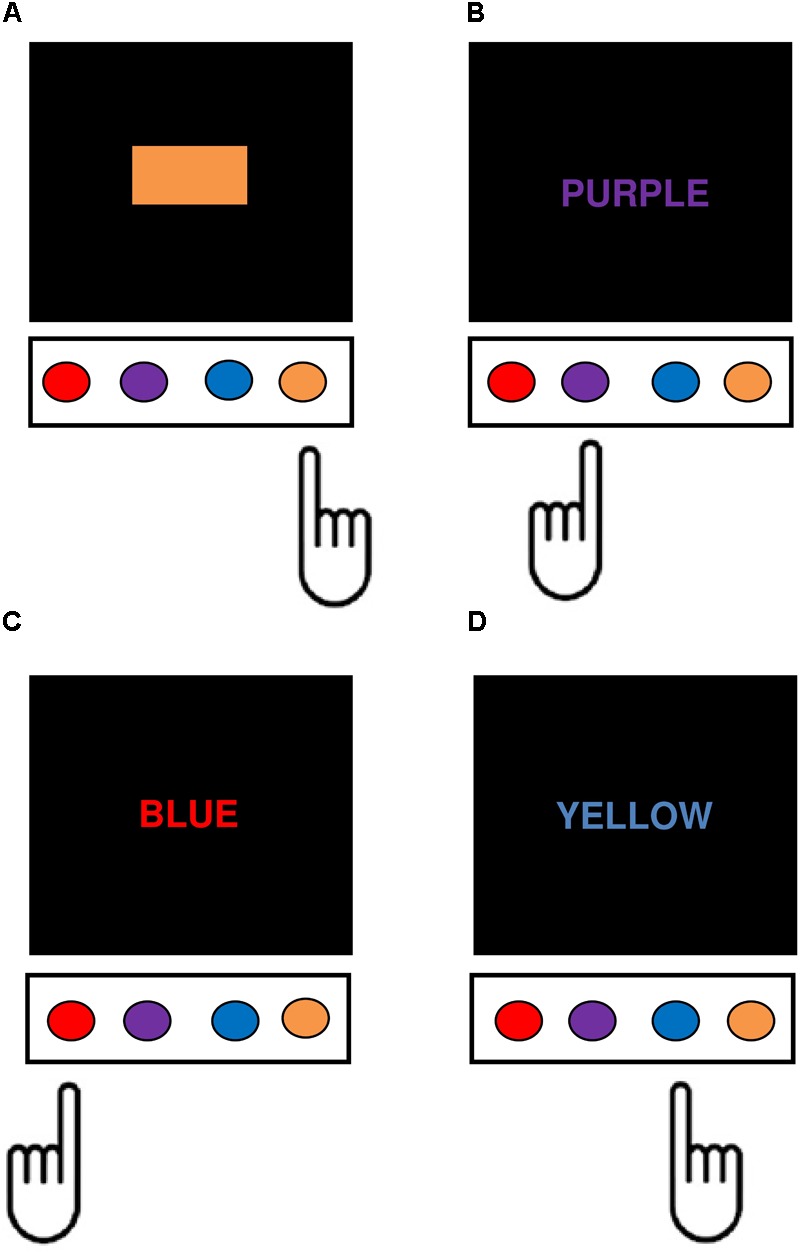
Depiction of the four main task conditions in the modified Stroop Task. In each condition, participants responded manually to the color identity of the task-relevant stimulus dimension by pushing the corresponding computer key. **(A)** Control (color identification) trials – participants responded to the color of the patches. **(B)** Congruent trials – participants responded to the text color, which corresponds semantically with the color word. **(C)** Incongruent response eligible – participants were required to ignore the color word and respond to the text color, which was mapped onto a conflicting manual response. **(D)** Incongruent response ineligible – participants were required to ignore the color word, which had no corresponding response, and instead respond to the text color in which the word was printed.

The current study used a modified version of the Stroop Color-Word Interference Test, adapted from the one previously described by Milham et al. (2001, 2003), programmed using PsychoPy V1.80.03 (Peirce, 2007, 2008) and administered on the same computer as the go/no-go and Simon tasks. There were four response keys corresponding with the colors red, purple, blue, and orange, mapped onto the keys ‘*D*,’ ‘*F*,’ ‘*K*,’ and ‘*L*,’ using color patches placed above each response key. Word stimuli consisted of eight uppercase color words with the width and height of each letter subtending 2° and 3° visual angle. There were four response eligible words, RED, PURPLE, BLUE, and ORANGE each with a corresponding response key, and four response ineligible words, BROWN, YELLOW, PINK, and GREEN, which had no corresponding response keys. Response eligible and ineligible color words were matched closely for log word frequency to within 0.05 units, as well as length in letters, number of syllables, and mean response times for speeded naming (Lund and Burgess, 1996; Balota et al., 2004). Response eligible words could appear in white (word reading condition), in a corresponding color (congruent condition), or in a non-corresponding color (incongruent response eligible condition), whereas response ineligible words could appear in one of the non-corresponding response eligible colors only (incongruent response ineligible condition). All color and color-word combinations were featured except for the word BROWN printed in blue, because the orthographic and phonemic overlap between the two color labels interferes with speeded naming (MacLeod, 1991). Color stimuli consisted of red, purple, blue, and orange color patches with length and height subtending 9° and 3° of visual angle, respectively (color naming condition) (**Figure 2**). The Stroop task consisted of four blocks: word-reading and color naming blocks consisting of 4 practice and 20 experimental trials each, and two experimental blocks consisting of 8 practice and 174 experimental trials in total (23 control, 74 congruent, 35 incongruent response eligible, and 42 incongruent response ineligible trials). The sequence for each trial began with presentation of the stimulus at central fixation until the participant responded. A 1000 ms inter-stimulus interval consisting of a blank screen then preceded the beginning of the next trial. The dependent variable was the differences in mean reaction time on incongruent response ineligible compared to control (i.e., color identification) trials (Stroop SS-conflict).

##### Modified flanker task

**FIGURE 3:**
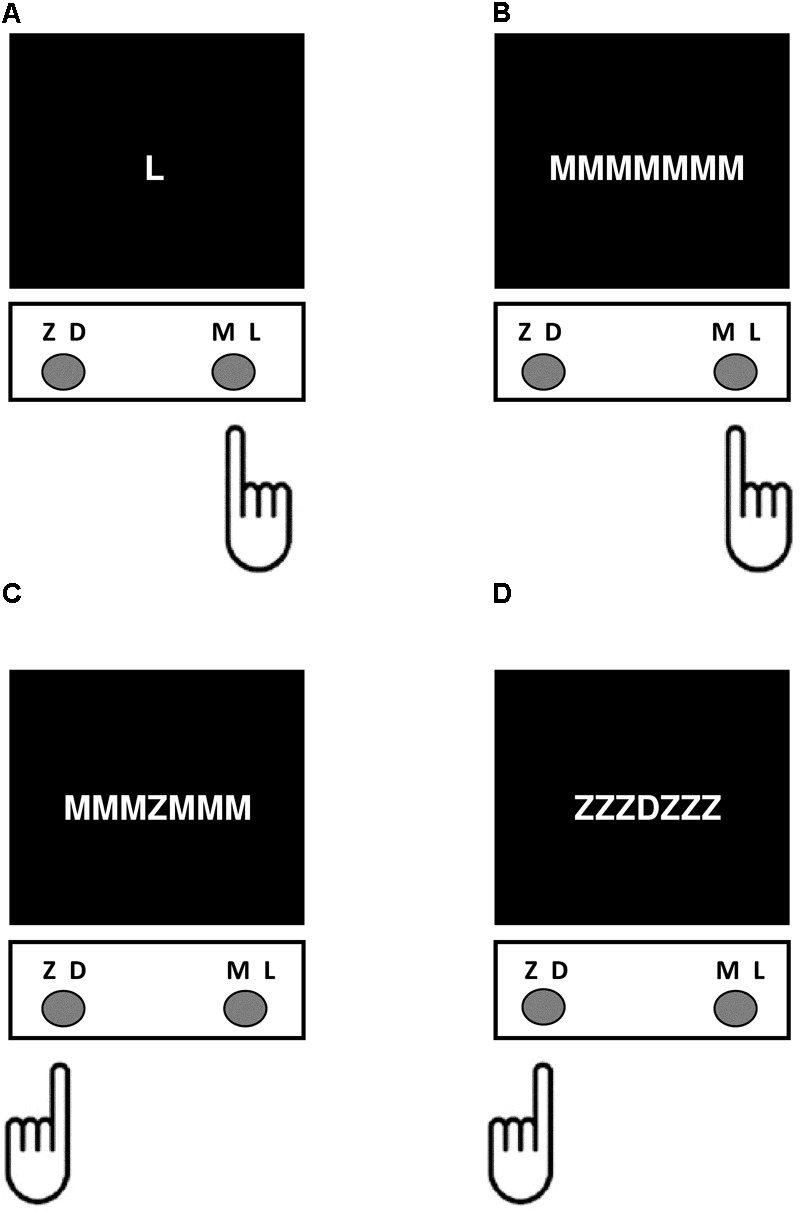
Depiction of the different trial conditions in the Flanker Task. For each trial type, participants responded by pressing the button on the keyboard corresponding to the identity of the letter appearing at central fixation. **(A)** Control condition – the target letter appeared by itself. **(B)** Congruent condition – the target letter appeared with three congruent flankers on either side. **(C)** Incongruent response incompatible condition – the letters flanking the target were mapped onto a conflicting response. **(D)** Incongruent response compatible condition – the incongruent flanking letters were mapped onto the same response.

The current study used a modified version of the Flanker task like that described by van Veen et al. (2001) programmed using PsychoPy V1.80.03 (Peirce, 2007, 2008) and administered on the same computer as the go/no-go, Simon, and Stroop tasks. Task stimuli consisted of four white letters ‘Z,’ ‘D,’ ‘M,’ and ‘L’ that appeared on a black background, with letter height subtending 2° and letter spacing subtending 0.004° of visual angle (Eriksen and Eriksen, 1974). These four letters were chosen because of close equivalence in global feature dissimilarity across all possible letter pairs (Eriksen, 1995; Mueller and Weidemann, 2012). The letters ‘Z,’ and ‘D’ were mapped onto the C key, and the letters ‘M,’ and ‘L’ were mapped onto the M key corresponding to left- and right-handed responses, respectively. On each trial type participants responded by pressing the button on the keyboard corresponding to the identity of the letter appearing at central fixation. Letter stimuli appeared in isolation (control condition), with flanking letters of the same identify (congruent condition), with incongruent flanking letters that were mapped onto the same response key (incongruent response compatible condition), or with incongruent flanking letters that were mapped onto the conflicting response (incongruent response incompatible condition) (**Figure 3**). There was a practice block of 10 trials followed by two experimental blocks consisting of a total of 160 trials, including 15 control, 50 congruent, 48 incongruent response compatible, and 47 incongruent response incompatible trials. Each trial began with presentation of a fixation cross at the central target location for 500 ms, followed by presentation of the stimulus until response. After the participant responded there was a 500 ms inter-stimulus interval before the next trial began. The dependent variable was the difference in mean reaction time on incongruent response compatible compared to congruent trials (Flanker SS-conflict).

##### Shape matching task

**FIGURE 4:**
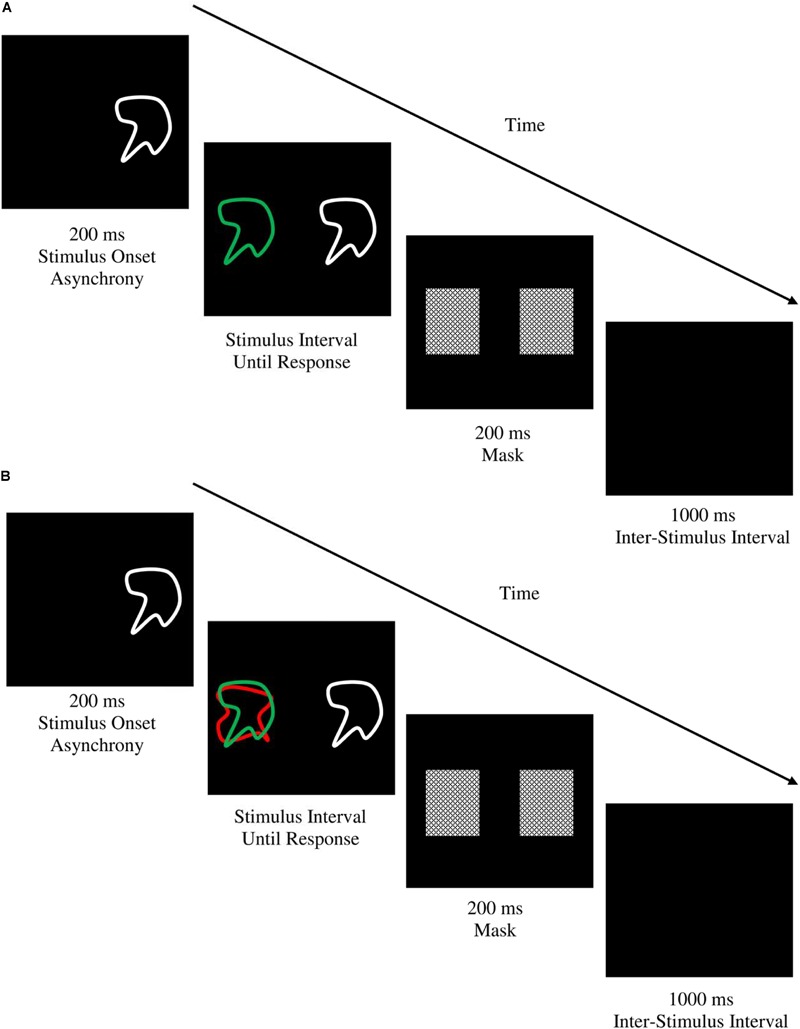
Trial sequence and timing for the **(A)** control and, **(B)** distractor conditions of the shape matching task. Each trial began with a 200 ms stimulus onset asynchrony with presentation of the comparison shape preceding the target and distractor stimuli so that participants first attended to the comparison shape before engaging selective attention processes for discriminating the target shape from the distractor, thereby enabling a more reliable measurement of stimulus-stimulus conflict (DeSchepper and Treisman, 1996). Stimuli were displayed until response after which a 200 ms mask was displayed to abolish any afterimages (Friedman and Miyake, 2004). A1000 ms inter-stimulus interval preceded the beginning of the next trial. In both examples above, the target and comparison shape are a match and participants would respond with a right-sided key press indicating ‘SAME.’

The shape matching task used in the current study was based on the task used by Friedman and Miyake (2004) to measure ‘Resistance to Distractor Interference.’ It was programmed using the same software and administered on the same computer as the previous SRC tasks. Task stimuli consisted of 20 abstract geometric shapes (10 target/comparison shapes and 10 distractor shapes) designed by the investigators, each subtending 10° of visual angle. The use of a separate set of shapes for distractor stimuli ensured there were no negative priming or facilitation effects due to interactions with target shapes (DeSchepper and Treisman, 1996). Target shapes were green in color and always appeared 7.5° to the left of central fixation. Distractor shapes were red in color and always appeared directly underlying the green target shapes. Comparison shapes were drawn from the same set as the target shapes but were white in color and always appeared 7.5° to the right of central fixation (see **Figure 4**). On each trial participants were required to indicate as quickly as possible using the appropriate manual response whether the target and comparison shapes were the same or different (the responses ‘Different’ and ‘Same’ were mapped onto the W and O keys). On control (no distractor) trials, the target and comparison shapes were presented on their own, whereas on distractor trials the target and comparison shapes were presented with the distractor shape, requiring participants to selectively attend to the green-colored target shape and ignore the red distractor shape to make the correct same/difference judgment. The sequence and timing of each trial is displayed in **Figure 4**. There were 14 practice trials, followed by two experimental blocks of 40 trials each. Conditions were balanced such that distractor trials were reproductions of control trials except for the presence of the underlying red distractor shape. The dependent variable was the difference in mean reaction time on distractor compared to control trials (shape matching SS-conflict).

### General Procedure

Participants were tested at varying locations, including their home, school, or at one of three research centers over a single 2-h session that was broken into 1-h blocks with additional short breaks in between tasks. Participants were seated comfortably in a location free from visual and auditory distractions. All stimuli were presented on a black background with viewing distance set at approximately 50 cm. An opaque cover was placed over the computer keyboard so that only the relevant response keys were visible for each task, with all the other keys disabled during the experiment. There was a 10-s break in between Blocks 1 and 2 for each task (as well as an additional break between Blocks 2 and 3 of Stop-It), after which participants pushed one of the response keys when they were ready to resume. Task order was fixed to control for any participant-by-order interaction effects, and was as follows, first block: Listening Recall, Counting Recall, Backward Digit Recall, Stroop, Flanker, and Simon tasks; second block: shape matching, and go/no-go tasks, and Stop-It. After completing Stop-It, participants were assessed using the Vocabulary, Matrix Reasoning, and Symbol Search subtests of the Wechsler Intelligence Scales for Children – Fourth Edition (WISC-IV) (Wechsler, 2003) and completed the Early Adolescent Temperament Questionnaire – Revised (EATQ-R) self-report form (Ellis and Rothbart, 2001). Parents or primary caregivers were asked to complete the Child Behavior Checklist/6 – 18 (CBCL), as well as the EATQ-R and the Behavior Rating Inventory of Executive Function (BRIEF) parent report forms (Gioia et al., 2000; Achenbach and Rescorla, 2001; Ellis and Rothbart, 2001). Participants and their primary caregivers were asked to complete the study questionnaires prior to the testing session and these were collected by the investigator after testing was complete. These measures were analyzed later as part of a different study.

### Statistical Procedures

#### Analysis of Missing Values, Normality, and Outliers

Data screening and preliminary analyses were conducted in SPSS. Of the total sample, 120 cases, constituting 88.2% of the data, had no missing data. Listening Recall scores were missing for 10 participants (7.4%), and four participants (∼3%) were missing Stroop and shape matching task data; three due to color blindness and the fourth on each task due to separate incidences of equipment failure. Finally, one participant (0.7%) was missing SSRT data due to equipment failure. Little’s (1988) Missing Completely at Random (MCAR) test was not significant [χ^2^(44) = 40.290, *p* = 0.631], indicating that the assumptions of MCAR were satisfied. For each participant the reaction time distributions for every SRC task condition were screened for outliers using a sequential fence procedure constructed using the upper and lower quartiles, defined as: *f*_Q_ = n/4 + (1/4), and a 2.2 multiplicative of the interquartile range (Hoaglin and Iglewicz, 1987). This method resulted in an average of 0.033% (0.019–0.036%) observations identified as outliers and removed across all participants, tasks, and conditions.

Skewness and kurtosis values were computed for the univariate distribution for each of the dependent variables and divided by their standard errors to assess normality (Tabachnick and Fidell, 2013). Variables with skew and kurtosis *Z* statistics exceeding the critical value of *Z* ± 1.96, *p* < 0.05 were considered to violate the assumption of univariate normality and these distributions were normalized using the appropriate transformation (see **Table 4**) (Tabachnick and Fidell, 2013). After removal of residual univariate outliers (Simon SR-conflict = 7; shape matching SS-conflict = 1; Stroop SS-conflict = 3; Flanker SS-conflict = 1) none of the variables exhibited significant skew. However, age (specified in number of months) and Flanker SS-conflict exhibited non-normal kurtosis. Tests of multivariate normality were conducted using the SPSS macro provided by DeCarlo (1997). Small’s (1980) test of multivariate skew was not significant [χ^2^(10) = 8.561, *p* = 0.574], however, Small’s test of multivariate kurtosis was significant [χ^2^(10) = 413.610, *p* < 0.001]. An omnibus test of multivariate normality, based on Small’s statistics was also significant [χ^2^(20) = 422.172, *p* < 0.001], indicating that the data set violated the assumption of multivariate normality (Small, 1980; DeCarlo, 1997). The variables were also assessed for multivariate outliers, with no cases exceeding the critical Mahalanobis Distance [χ^2^(10) = 29.588, *p* < 0.001] (Tabachnick and Fidell, 2013).

#### Calculation of Interference Effects on SRC Tasks

Interference effects for each participant were calculated by subtracting mean reaction time trials in the baseline condition from mean reaction time on incongruent trials. Control trials were used as the baseline in the Stroop and Simon tasks because congruity effects often result in response facilitation and faster reaction times on congruent trials (Kornblum et al., 1990; MacLeod, 1991; Lu and Proctor, 1995). The congruent condition was used as the baseline for the Flanker task because mean reaction time on control trials was slower than on incongruent response compatible trials, and this is also the standard approach for calculating the conflict effect on the Flanker and related Attention Network Test (van Veen et al., 2001; Fan et al., 2002; Rueda et al., 2004). SS-conflict was calculated for the Stroop task by subtracting mean reaction time on control (i.e., color identification) trials from mean reaction time on incongruent response ineligible trials. SS-conflict was calculated for the Flanker task by subtracting mean reaction time on congruent trials from mean reaction time on incongruent response compatible trials. The neurocognitive mechanism underlying response selection on the Stroop and Flanker tasks is considered to be behaviorally and neurobiologically distinct from the one implicated in non-selective motoric stopping on the stop signal and go/no-go tasks (Scheres et al., 2003; Chambers et al., 2007, 2009; Egner et al., 2007). However, SR-conflict on the Stroop and Flanker tasks was calculated for the purposes of estimating the proportion of the interference effect on these tasks attributable to the two types of conflict. SR-conflict was calculated for the Stroop task by subtracting mean reaction time on incongruent response ineligible trials from mean reaction time on incongruent response eligible trials. SR-conflict was calculated for the Flanker task by subtracting mean reaction time on incongruent response compatible trials from mean reaction time on incongruent response incompatible trials.

#### Model Estimation

All measurement and structural regression models were estimated in Mplus 7.2 using the covariance matrix with Full Information Maximum Likelihood, which uses the Expectation Maximization algorithm to account for missing data (Muthén and Muthén, 1998-2012). The Maximum Likelihood estimator is considered to be fairly robust to minor departures from multivariate normality (Byrne, 2012). However, model estimation in small samples using variables with non-normal kurtosis can bias the χ^2^ statistic toward significance (Type II error), as well as attenuating standard errors for the model parameters estimates biasing them toward significance (Type I error) (Enders, 2010). Therefore, the Bollen-Stine Bootstrap procedure was used with 25,000 posterior draws in order to calculate an adjusted probability value for the χ^2^ test of model fit, as well as bootstrapped standard errors with 95% confidence intervals for the model parameter estimates (Muthén and Muthén, 1998-2012; Enders, 2010). Congeneric measurement models were specified with target factor loadings and factor intercorrelations freely estimated, and non-target indicator cross-loadings and error covariances constrained to zero (Anderson and Gerbing, 1988; Hair et al., 2006). Latent variable scaling was performed using the fixed factor method (Muthén and Muthén, 1998-2012; Byrne, 2012).

Parameter estimates for models with a case-to-parameter ratio (*N*:*q*) of less than 10:1 can be biased and untrustworthy (Kline, 2016). A two-step Factor Score Regression method was therefore used to reduce model complexity (Devlieger et al., 2016; Devlieger and Rosseel, 2017). Factor score estimates were first generated for the WMC factor in a separate CFA model and were then used as a single indicator latent variable in the hierarchical structural regression model. The Factor Score Regression method results in unbiased parameter estimates in structural regression models when the factor score estimates are used as an exogenous (i.e., independent) latent variable (Devlieger et al., 2016; Devlieger and Rosseel, 2017).

Model fit was assessed using a combination of absolute and incremental fit indices, including the chi square (χ^2^) test statistic; the Root Mean Square Error of Approximation (RMSEA); the Comparative Fit Index (CFI); and the Standardized Root Mean Square Residual (SRMR) (Byrne, 2012; Kline, 2016). The χ^2^ test statistic has been found to be the most sensitive fit index for identifying model misspecification and was therefore referred to first in order to adjudge model fit (Marsh et al., 2004). Generally accepted cut-off criteria for the RMSEA are: 𝜀 < 0.05 = close approximate fit, 𝜀 0.05–0.08 = reasonable approximate fit; 𝜀 > 0.1 = poor fit (Kline, 2011). However, models with few degrees of freedom (*df*) and small sample sizes are underpowered to detect model misspecification using the χ^2^ test statistic and RMSEA (Kenny et al., 2015). Therefore, model fit was also evaluated by examining the significance and strength of individual parameter estimates (Kline, 2016). A cut-off criterion of >0.90 (Bentler and Bonett, 1980; Browne and Cudeck, 1993; Hair et al., 2006) was used for the CFI, because the commonly used cut-off criterion of >0.95 often leads to inflated Type II error rates and is not appropriate for use in samples that are not asymptotically robust, and models expected to have low factor loadings (Hu and Bentler, 1999; Marsh et al., 2004; Bagozzi and Yi, 2012; Heene et al., 2012). Values below 0.08 were considered to indicate acceptable fit for the SRMR (Hu and Bentler, 1999). The chi-square difference test (Δχ^2^) was used for comparing the relative fit of competing nested models (Marsh et al., 2004).

## Results

### Task Results

**FIGURE 5:**
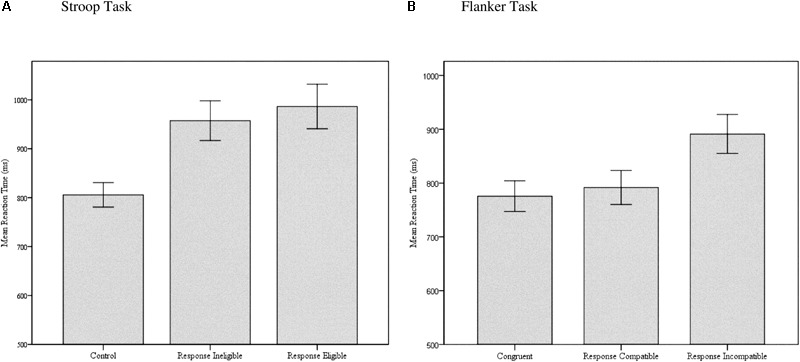
Bar graphs of mean reaction times on the **(A)** Stroop and **(B)** Flanker task for the different task conditions, demonstrating the relative contribution of SS-conflict and SR-conflict to the overall interference effect. Error bars represent 95% confidence intervals.

Results from the AWMA subtests are displayed in **Table 2** and were indicative of a participant sample with slightly higher than average WMC compared with the age-related population, along with a slight truncation in score ranges with no individuals scoring below a standard score of 70 (*Extremely Low*). Results from the various conditions of the SRC tasks are displayed in **Table 3** and reveal good to excellent internal consistency reliability. In general, the expected pattern of differences across task conditions were observed with slower mean reaction times on incongruent compared to congruent and control trials, and slower mean reaction times on response eligible and response incompatible trials compared to response ineligible and response compatible trials of the Stroop and Flanker tasks. As displayed in **Figure 5A**, 84% of the standard Stroop interference effect of 181 ms obtained between control and incongruent response eligible trials was attributable to SS-conflict (152 ms), whereas SR-conflict contributed only 16% of the total interference effect (29 ms). In contrast, **Figure 5B** shows that 86% of the 116 ms total interference effect in the Flanker task was attributable to SR-conflict (99 ms), whereas only 14% was attributable to SS-conflict (16 ms).

**Table 2:** Descriptive statistics for standard scores from the automated working memory assessment.

		95% CI			
Subtest Standard Score	*M*	LL	UL	*SD*	Range
Listening Recall^1^	109.324	106.336	112.311	16.944	77.9–145.7
Counting Recall	105.933	103.627	108.239	13.599	76.6–141.0
Backward Digit Recall	105.219	102.542	107.897	15.788	77.1–145.0
Verbal Working Memory Composite^1^	107.778	105.197	110.359	14.639	76.0–139.0

*N = 136. M, mean; CI, confidence interval; LL, lower limit; UL, upper limit; SD, standard deviation. ^*1*^n = 126*.

**Table 3:** Descriptive statistics for the different stimulus-response compatibility task conditions^1^.

Task	Condition	*M*	95% CI		*SD*	*Range*	Reliability^2^
			*LL*	*UL*			
Stroop	Control	806	781	831	145	514–1300	0.89
	Congruent	808	780	836	163	541–1276	0.96
	Ineligible	957	917	998	236	546–2061	0.94
	Eligible	986	941	1032	264	570–1999	0.92
Flanker	Control	865	825	904	233	488–1601	0.85
	Congruent	776	747	804	168	496–1203	0.95
	Compatible	792	760	823	187	480–1342	0.96
	Incompatible	89	855	927	214	523–1575	0.94
Simon	Control	607	583	631	143	398–1237	0.81
	Congruent	608	587	630	127	392–1043	0.90
	Incongruent	617	599	635	108	414–1120	0.90
Shape matching	Control	855	816	895	230	483–2121	0.91
	Distractor	1088	1027	1150	360	613–353	0.94

*N = 136. M, mean; CI, confidence interval; LL, lower limit; UL, upper limit; SD, standard deviation. ^*1*^All figures are in milliseconds (ms); ^*2*^internal consistency reliability calculated using Cronbach’s alpha*.

Repeated measures ANOVA with Greenhouse-Geisser correction for within-subject comparisons was significant for the Stroop [*F*(1.505,197.127) = 138.608, *p* < 0.001, $\eta_{p}^{2}$ = 0.514] and Flanker [*F*(2.118,285.970) = 70.754, *p* < 0.001, $\eta_{p}^{2}$ = 0.344] tasks, but not the Simon task [*F*(1.585,213.967) = 1.334, *p* = 0.263]. Tests of within-subjects contrasts on the Stroop task indicated that mean reaction time was not significantly different between control and congruent trials [*F*(1,131) = 0.110, *p* = 0.740]; but was significantly slower on incongruent response ineligible compared to congruent trials [*F*(1,131) = 209.669, *p* < 0.001, $\eta_{p}^{2}$ = 0.615]; and incongruent response eligible compared to incongruent response ineligible trials [*F*(1,135) = 12.944, *p* < 0.001, $\eta_{p}^{2}$ = 0.090]. Tests of within-subjects contrasts on the Flanker task indicated that mean reaction time was significantly slower on control compared to congruent trials [*F*(1,135) = 77.206, *p* < 0.001, $\eta_{p}^{2}$ = 0.364]; incongruent response compatible compared to congruent [*F*(1,135) = 9.617, *p* < 0.01, $\eta_{p}^{2}$ = 0.067]; and incongruent response incompatible compared to incongruent response compatible [*F*(1,135) = 184.834, *p* < 0.001, $\eta_{p}^{2}$ = 0.578]. Data from the Simon task revealed only a small difference in mean reaction times between all three conditions, with an average of only 9 ms interference effect obtained on incongruent compared to control trials. Results obtained from the shape matching task revealed a robust interference effect (233 ms) when comparing mean reaction times on the control and distractor conditions [*F*(1,131) = 237.903, *p* < 0.001, $\eta_{p}^{2}$ = 0.645]. Given the higher ratio of male compared to female participants in the current sample, a MANOVA was conducted to determine if there were any sex-related differences across any of the dependent variables. The test of multivariate effects was not significant [Wilks’ λ = 0.941, *F*(10,98) = 0.609, *p* = 0.803, observed power = 0.3], suggesting no statistically significant differences between male and female participants across the dependent variables in the study, although observed power was low.

Descriptive statistics for the dependent measures used as latent variable indicators in the current study are displayed in **Table 4**. The internal consistency reliabilities of the tasks used in the current study were generally good to excellent (Streiner, 2003). This was surprising given the low reliabilities observed in previous studies examining similar constructs, as well as the lower internal consistency reliabilities generally associated with difference scores calculated across task conditions (Cohen and Cohen, 1983; Miyake et al., 2000; Friedman and Miyake, 2004; Stahl et al., 2014). Strong psychometric properties for the indicator variables is a first step toward establishing the construct validity of the subsequent measurement model in SEM (Hair et al., 2006). Zero order correlations between the variables are displayed in **Table 5**. The pattern of correlations found in the current study was consistent with the low to modest linear relationships found in previous studies of higher-order cognitive abilities, particularly in developmental samples, and is reflective of the task impurity problem (Miyake et al., 2000; Friedman and Miyake, 2004; St Clair-Thompson and Gathercole, 2006; Wiebe et al., 2008). SSRT was weakly and negatively correlated age, as has been observed in previous research (Williams et al., 1999). The absence of age-related correlations in the other variables likely reflected the use of a developmental sample with a constrained age range. To remove age-related variance, SSRT was regressed onto age and the unstandardized residuals were used as the indicator variable in subsequent analyses. Minimal change was observed in the intercorrelations of SSRT with the other variables after age-related variance had been partialled out. The strength of the correlations increased marginally with Backward Digit Recall (*r* = -0.294, *p* = 0.001), Simon SR-conflict (*r* = -0.172, *p* = 0.052), and shape matching SS-conflict (*r* = 0.267, *p* = 0.002), and slightly decreased with Counting Recall (*r* = -0.257, *p* = 0.003), and no-go commission errors (*r* = 0.376, *p* < 0.001). The correlation with Listening Recall was unchanged, and SSRT was still not significantly correlated with Stroop and Flanker SS-conflict.

**Table 4:** Descriptive statistics for the variables used in the current study.

Variable	*M*	95% CI		*SD*	Range	Skewness	Kurtosis	Reliability	*n*
		*LL*	*UL*						
Age	142.460	141	144	8	132–156	1.269	-3.584^∗^		136
LR^1^	15.476	14.758	16.195	4.075	8–27	0.894	-1.150	0.88^3^	126
CR	22.625	21.868	23.382	4.464	13–35	0.298	1.162	0.83^3^	136
BDR^1^	16.463	15.566	17.360	5.289	7–30	1.332	-1.504	0.86^3^	136
SSRT^2^	281	267.958	293.221	74.203	119–579	0.110	1.372	0.94^4^	135
No-Go^1^	13.550	12.455	14.648	6.466	3–35	1.038	-0.908	0.82^5^	136
Simon^1^	16	5	27	63	-224–142	0.587	0.816	0.58^5^	129
SMT^2^	235	205	265	172	11–1232	0.052	1.412	0.84^5^	131
SSC^1^	144	123	165	122	-72–571	1.65	1.582	0.86^5^	129
FSC^1^	17	7	27	61	-91–252	0.469	1.795^∗^	0.71^5^	135

*N = 136. M, mean; CI, confidence interval; LL, lower limit; UL, upper limit; SD, standard deviation; n = size of subsample. LR, Listening Recall; CR, Counting Recall; BDR, Backward Digit Recall; SSRT, Stop-Signal Reaction Time; No-Go, Commission errors on Go/No-Go task; Simon, Simon SR-conflict; SMT, Shape matching SS-conflict; SSC, Stroop SS-conflict; FSC, Flanker SS-conflict. ^*1*^Variable transformed using a square root transformation; ^*2*^variable transformed using a logarithmic transformation; ^*3*^test–retest reliability as reported in the AWMA Manual; ^*4*^ internal consistency reliability computed using Split Half correlation; ^*5*^internal consistency reliability estimate computed using Split Half correlation with Spearman–Brown correction. ^∗^p < 0.05*.

**Table 5:** Intercorrelations amongst the variables.

	1	2	3	4	5	6	7	8	9
(1) Age									
(2) LR	0.031								
(3) CR	0.062	0.499***							
(4) BDR	0.051	0.495***	0.482***						
(5) SSRT	-0.219*	-0.346***	-0.265**	-0.287**					
(6) No-Go	-0.085	-0.141	-0.232**	-0.339***	0.386***				
(7) Simon	0.034	-0.117	-0.265**	-0.292**	0.161^	0.160^			
(8) SMT	-0.008	-0.215*	-0.205*	-0.113	0.262**	0.054	0.108		
(9) SSC	-0.080	-0.046	-0.139	-0.146^	0.087	-0.105	0.043	0.299**	
(10) FSC	0.019	-0.068	0.034	0.079	0.013	-0.033	0.031	0.091	0.196*

*N = 136. LR, Listening Recall; CR, Counting Recall; BDR, Backward Digit Recall; SSRT, Stop-Signal Reaction Time; No-Go, Commission errors on Go/No-Go task; Simon, Simon SR-conflict; SMT, Shape matching SS-conflict; SSC, Stroop SS-conflict; FSC, Flanker SS-conflict; ^∗∗∗^p < 0.001, ^∗∗^p < 0.01, ^∗^p < 0.05, ˆp < 0.10.*

### Model Results

#### Measurement Model Results

**FIGURE 6:**
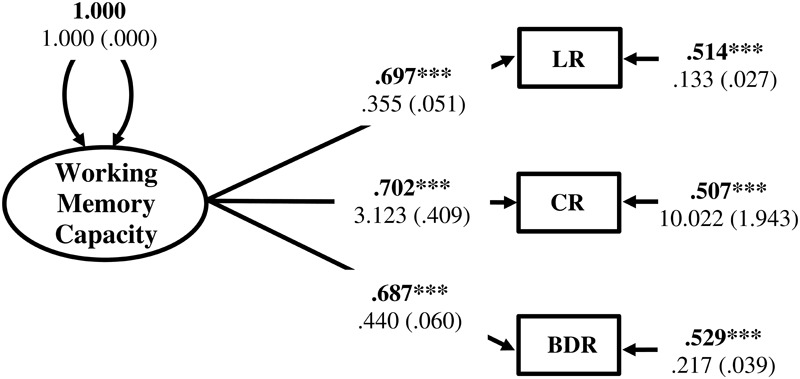
Measurement model for the Working Memory Capacity (WMC) factor. LR, Listening Recall; CR, Counting Recall; BDR, Backward Digit Recall. Fully standardized estimates are in bold type face. Numbers below are unstandardized parameter estimates with bootstrapped standard errors in brackets. ^∗∗∗^*p* < 0.001.

Working memory capacity factor score estimates were generated in Mplus from the CFA of Listening Recall, Counting Recall, and Backward Digit Recall raw scores using the factor score regression method (Muthén and Muthén, 1998-2012; Devlieger et al., 2016). Results of the CFA model for the WMC factor are displayed in **Figure 6**. Construct reliability of the WMC factor was evaluated by calculation of the *H* index (varying from 0 – 1) using the standardized loading estimates (Hancock and Mueller, 2001). The *H* index for the WMC factor was 0.74 indicating that it had adequate construct reliability and was likely to be replicable across studies using the same indicators (Hancock and Mueller, 2001; Rodriguez et al., 2016). Factor score determinacy was also relatively high (ρ = 0.859), suggesting that the factor score estimates provided fairly accurate measures of individual differences with respect to the WMC latent variable (Grice, 2001; DiStefano et al., 2009). In addition, regression coefficients are unbiased when factor score estimates, generated using the factor score regression method, are the independent variables in a structural model (Devlieger et al., 2016; Devlieger and Rosseel, 2017). However, to reduce potential error in parameter estimates, the factor score estimates were entered into the subsequent structural regression model as a single indicator latent variable with error variance fixed to reflect the unreliability of the factor score estimates [*q* = (1 - validity)^∗^Var(indicator)] (Bollen, 1989). SSRT, no-go commission errors, and Simon SR-conflict, as well as shape matching SS-conflict, Stroop SS-conflict, and Flanker SS-conflict were also entered into the model as indicators of the Response Inhibition and Attentional Inhibition factors, respectively.

Fit statistics for the competing measurement models are displayed in **Table 6**. The initial correlated three-factor model (Model 1) provided a good fit to the data. In particular, the χ^2^ test statistic and RMSEA point estimate indicated that the null hypotheses of exact and close approximate fit to the observed covariance matrix could not be rejected (MacCallum et al., 1996; Kline, 2016). The only target loading that was not significant was Flanker SS-conflict on the Attentional Inhibition factor (λ = 0.167, *p* = 0.233). This result was not surprising given the very small proportion of variance in overall interference effect on the Flanker task attributable to SS-conflict. However, dropping this item from the model caused estimation problems due to local under-identification of the Attentional Inhibition latent variable (Bollen, 1989). Neither imposing tau equivalence nor constraining error variances to be equal for the two indicator variables provided a solution for model identification (Hair et al., 2006). Therefore, the Flanker SS-conflict indicator variable was retained for model identification, which also enabled factor loadings and error variances for the Attentional Inhibition factor to be freely estimated (Bollen, 1989; Hair et al., 2006).

**Table 6:** Summary of fit statistics for the competing confirmatory factor analysis models.

	Model	*df*	χ^2^	*p*	RMSEA (90% CI)	SRMR	CFI	AIC
1	Three component	12	13.262	0.384	0.028 (0.000-0.094)	0.048	0.982	2862.640
2	Null/baseline A	21	90.565	<0.001	0.156 (0.124-0.190)	0.151	0.000	2921.942
3	Constrained three-factor A (correlation between RI and AI constrained to 1)	13	25.308	0.036	0.083 (0.032-0.132)	0.069	0.823	2872.685
4	Constrained three-factor B (correlation between RI and AI constrained to 0)	13	15.987	0.290	0.041 (0.000-0.099)	0.062	0.957	2863.364
5	Constrained three-factor C (correlation between WMC and AI constrained to 0)	14	19.233	0.199	0.052 (0.000-0.105)	0.071	0.925	2864.610
6	Constrained three-factor D (correlation between WMC and RI constrained to 0)	14	42.823	0.001	0.123 (0.082-0.166)	0.109	0.586	2888.201
7	Two factor (RI and AI with no WMC)	8	7.954	0.485	0.000 (0.000-0.100)	0.048	1.00	2541.280
8	Null/baseline B	15	55.239	<0.001	0.140 (0.102-0.181)	0.126	0.000	2574.565
9	Constrained two factor A (correlation between RI and AI constrained to 1)	9	20.289	0.033	0.096 (0.039-0.152)	0.075	0.719	2551.615
10	Constrained two factor B (correlation between RI and AI constrained to 0)	9	12.314	0.269	0.052 (0.000-0.117)	0.061	0.918	2543.640

*df, degress of freedom; χ^*2*^, Chi square value for test of model fit using Full Information Maximum Likelihood estimation; p, significance value of the chi square test statistic; RMSEA, Root Mean Square Error of Approximation; CI, Confidence Interval; SRMR, Standardized Root Mean Square Residual; CFI, Comparative Fit Index; AIC, Akaike Information Criterion. WMC, Working Memory Capacity; RI, Response Inhibition; AI, Attentional Inhibition*.

Although the amount of variance explained in the remaining indicator variables was in some cases small, this is regarded as typical for complex cognitive functions due to the task impurity problem (Friedman and Miyake, 2004). More importantly, all measured variables loaded on their hypothesized factor consistent with the proposed theoretical framework, providing evidence for construct validity (Hair et al., 2006). However, given the modest zero order correlations amongst the observed variables, an initial consideration was the factorability of the covariance matrix and whether the theoretical model provided a significant improvement in fit over the null model with all correlations constrained to zero (Byrne, 2012). The null model was estimated and provided a significantly worse fit to the data than the measurement model [Δχ^2^(9) = 77.303, *p* < 0.05], suggesting that there was sufficient covariance in the data to identify the hypothesized Response Inhibition and Attentional Inhibition factors, along with WMC. However, the *H* index was computed for the Response Inhibition (*H* = 0.56) and Attentional Inhibition (*H* = 0.62) factors and indicated less than adequate construct reliability (*H* < 0.70) (Hancock and Mueller, 2001; Rodriguez et al., 2016).

Constraining factor intercorrelations to zero or one provides a powerful statistical test of comparative fit for the theoretical measurement model relative to more constrained nested models (Anderson and Gerbing, 1988; Kline, 2016). Constraining the intercorrelations between Response Inhibition and WMC, and separately Attentional Inhibition with WMC, to one resulted in model non-convergence, indicating model misspecification. Constraining the intercorrelation between Response Inhibition and Attentional Inhibition to one (Model 3) resulted in a significant decrement in model fit indicating that these two inhibitory abilities are best thought of as distinct cognitive constructs. Conversely, constraining this factor intercorrelation to zero (Model 4) did not result in significantly worse fit [Δ*χ*^2^(1) = 2.725, *p* > 0.05], suggesting a more parsimonious representation of the data.

**FIGURE 7:**
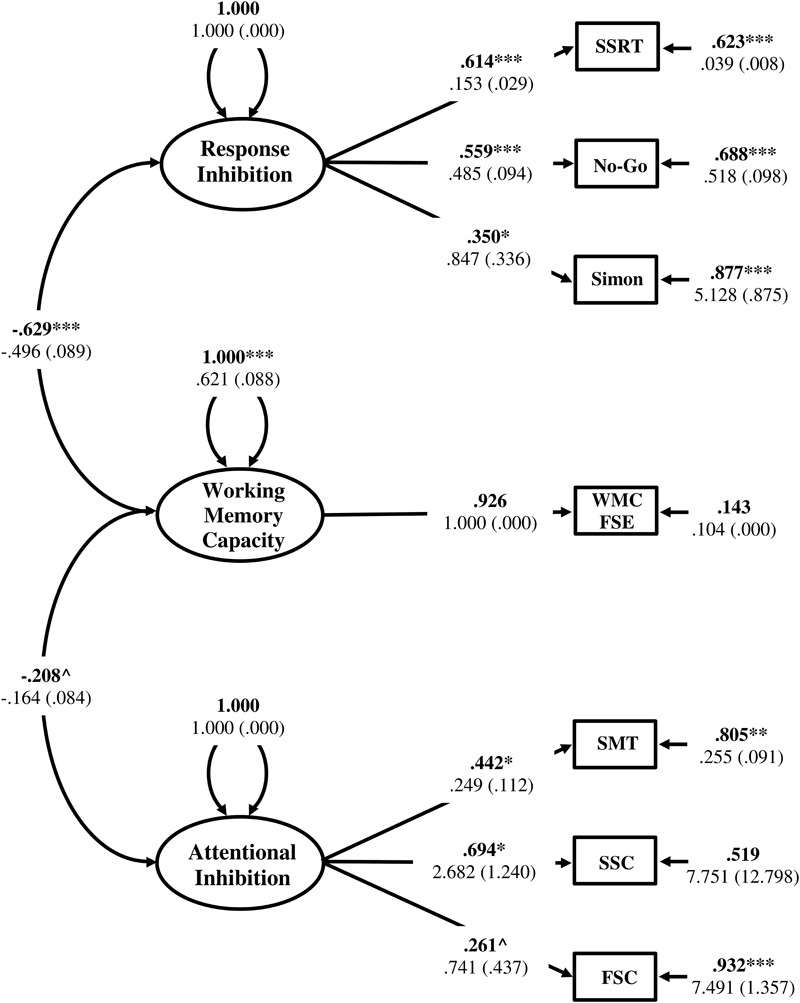
Measurement model of inhibitory control with three, empirically distinct factors corresponding to WMC, Response Inhibition, and Attentional Inhibition. WMC FSE, Working Memory Capacity Factor Scores Estimates; SSRT, Stop-Signal Reaction Time; No-Go, Commission errors on Go/No-Go task; Simon, Simon SR-conflict; SMT, Shape matching SS-conflict; SSC, Stroop SS-conflict; FSC, Flanker SS-conflict. Fully standardized estimates are in bold type face. Numbers below are unstandardized parameter estimates with bootstrapped standard errors in brackets. ^∗∗∗^*p* < 0.001, ^∗∗^*p* < 0.01, ^∗^*p* < 0.05, ˆ*p* < 0.1.

Applying a parameter constraint of zero on the factor intercorrelation between WMC and Attentional Inhibition (Model 5) resulted in a poorer overall model fit that was not significantly worse [Δχ^2^(1) = 3.246, *p* > 0.05], but resulted in local under-identification of the Attentional Inhibition factor (shape matching SS-conflict λ = 0.406, *p* = 0.076; Stroop SS-conflict λ = 0.735, *p* = 0.051; Flanker SS-conflict λ = 0.271, *p* = 0.070). Therefore, this model was not retained. Constraining the intercorrelation between WMC and Response Inhibition to zero (Model 6) resulted in a significantly poorer model fit [Δχ^2^(1) = 26.836, *p* < 0.05]. Therefore, the model with Response Inhibition and Attentional Inhibition specified as uncorrelated factors (Model 4) provided the best representation of the data. This model is displayed in **Figure 7**.

The Response Inhibition and Attentional Inhibition factors were also modeled without the WMC factor included (Model 7). The 95% confidence interval of the standardized factor intercorrelation between Response Inhibition and Attentional Inhibition did not contain one or zero (ϕ = 0.322, 95%*CI* = 0.055–0.590, *p* = 0.060) and was similar in strength to that observed in the three-factor model (ϕ = 0.343; 95%*CI* = 0.059–0.627, *p* = 0.070). The results in **Table 6** show that this correlated two-factor model provided a good fit to the data that was significantly better than a corresponding null model [Model 8; Δχ^2^(7) = 47.285, *p* < 0.05], as well as nested models with the factor intercorrelation constrained to one [Model 9; Δχ^2^(1) = 12.335, *p* < 0.05] and zero [Model 10; Δχ^2^(1) = 4.360, *p* < 0.05]. Thus, Response Inhibition and Attentional Inhibition could not be represented as independent constructs when WMC was not included in the model.

#### Structural Regression Model Results

**FIGURE 8:**
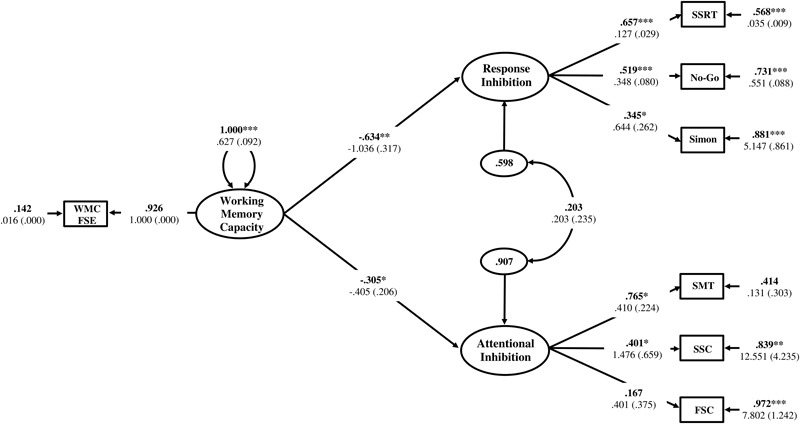
Hierarchical model of inhibitory control with the Response Inhibition and Attentional Inhibition factors regressed onto WMC specified as a higher-order factor. WMC FSE, Working Memory Capacity Factor Scores Estimates; SSRT, Stop-Signal Reaction Time; No-Go, Commission errors on Go/No-Go task; Simon, Simon SR-conflict; SMT, Shape matching SS-conflict; SSC, Stroop SS-conflict; FSC, Flanker SS-conflict. Fully standardized estimates are in bold type face. Numbers below are unstandardized parameter estimates with bootstrapped standard errors in brackets. ^∗∗∗^*p* < 0.001, ^∗∗^*p* < 0.01, ^∗^*p* < 0.05.

**FIGURE 9:**
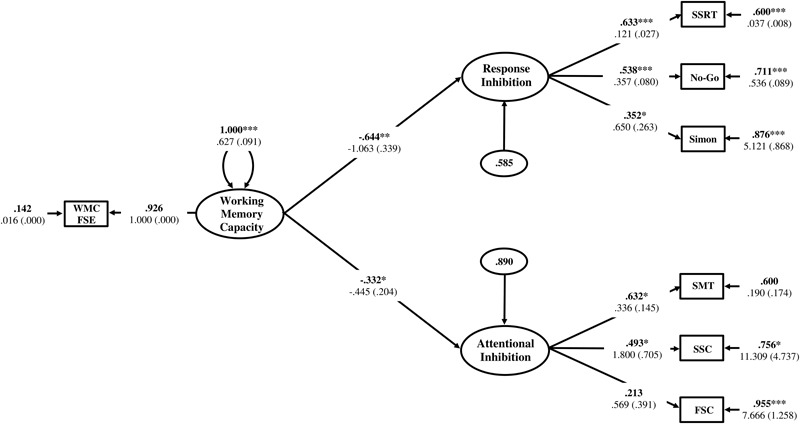
Hierarchical model of inhibitory control with the residual correlation between Response Inhibition and Attentional Inhibition factors constrained to zero. WMC FSE, Working Memory Capacity Factor Scores Estimates; SSRT, Stop-Signal Reaction Time; No-Go, Commission errors on Go/No-Go task; Simon, Simon SR-conflict; SMT, Shape matching SS-conflict; SSC, Stroop SS-conflict; FSC, Flanker SS-conflict. Fully standardized estimates are in bold type face. Numbers below are unstandardized parameter estimates with bootstrapped standard errors in brackets. ^∗∗∗^*p* < 0.001, ^∗∗^*p* < 0.01, ^∗^*p* < 0.05.

The Response Inhibition and Attentional Inhibition factors were regressed onto the single-indicator WMC latent variable to test the first hypothesis that these two constructs would exhibit a shared statistical dependence on WMC as a higher-order factor. The residual correlation between Response Inhibition and Attentional Inhibition was freely estimated to determine if there was a decrease in the empirical association between these constructs after the variance shared with WMC was accounted for. The structural regression model is displayed in **Figure 8**. The regression coefficients of Response Inhibition (γ = -0.634, *p* = 0.001) and Attentional Inhibition (γ = -0.305, *p* = 0.049) were both statistically significant and indicated that the efficiency of inhibitory control increased concomitantly with higher levels of WMC. The residual correlation between Response Inhibition and Attentional Inhibition was not statistically significant (ψ = 0.203, *p* = 0.387), suggesting that their previous marginally significant association (ϕ = 0.343, *p* = 0.070), was explained by their shared empirical association with WMC. Constraining this residual correlation to zero provided a more parsimonious model (**Figure 9**) that did not lead to a significant decrement in model fit [χ^2^(13) = 14.117, *p* = 0.409; RMSEA = 0.025; 90%CI = 0. 000–0.091; CFI = 0.984; SRMR = 0.051; Δχ^2^(1) = 0.855, *p* > 0.05], and was thus retained as the final model. In this final model, WMC explained 41% (γ = -0.644; γ^2^ = 0.415, *p* = 0.002) and 11% (γ = -0.332; γ^2^ = 0.110, *p* = 0.029) of the variance in the Response Inhibition and Attentional Inhibition factors, respectively.

A theoretically plausible equivalent model was generated using Lee-Herschberger replacing rules, which assists in determining whether the theoretical structural regression model provides the optimal representation of the data compared to an alternative model with equivalent overall fit (Lee and Hershberger, 1990; MacCallum and Austin, 2000). Equivalent models can be evaluated with respect to the theoretical plausibility and empirical fit of the individual parameter estimates (Kline, 2016). WMC was regressed onto Attentional Inhibition to reflect the theory that ‘interference control’ functions as a sensory gating mechanism that protects working memory from interference (Bjorklund and Harnishfeger, 1990; Lustig et al., 2007). The regression coefficient was significant (γ = -0.305, *p* = 0.014) explaining 9.3% of the variance in WMC. However, constraining the non-significant correlation between Attentional Inhibition and Response Inhibition increased the regression coefficient (γ = -0.332, *p* = 0.008), explaining 11% of the variance in WMC.

As this observed effect was comparable to the regression of Attentional Inhibition on WMC, tests of directional dependence were conducted to determine which of the two was best interpreted as the exogenous variable (von Eye and Wiedermann, 2014). Factor score estimates for the WMC and Attentional Inhibition factors were compared with respect to their third- and fourth-order moments. The sample distribution of factor score estimates for the WMC [γ = 1.178, *p* > 0.05; κ = 0.421, *p* > 0.05; *W*(136) = 0.992, *p* = 0.684] and Attentional Inhibition [γ = 2.014, *p* > 0.05; κ = 1.688, *p* > 0.05; *W*(136) = 0.982, *p* = 0.069] latent variables did not deviate from normality as revealed by skewness and kurtosis coefficients and Shapiro–Wilk tests. Similarly, neither the residuals of WMC factors score estimates regressed onto the Attentional Inhibition factor score estimates [γ_𝜀_ = 0.875, *W*(136) = 0.989, *p* = 0.327], nor the reverse [γ_𝜀_ = 1.274; *W*(136) = 0.991, *p* = 0.505] were significantly skewed. Direction of dependence cannot be determined from third- and fourth-order moments when the distributions of factor scores estimates are univariate normal (von Eye and Wiedermann, 2014). In addition, the validity of the factor score estimates for the Attentional Inhibition factor were less than recommended (ρ = 0.722), suggesting error in the estimates (Grice, 2001). This was also evident in the lack of correlational preserving between the factor score estimates (*r* = -0.488, *p* < 0.001) compared to the factor intercorrelation in the full latent model (ϕ = -0.349, *p* = 0.015) (Grice, 2001; DiStefano et al., 2009). Thus, evidence for directional dependence was equivocal, and the theoretical structural regression model was retained based on *a priori* predictions (von Eye and Wiedermann, 2014).

## Discussion

The proposed hierarchical model of inhibitory control was partially supported by the results of structural equation modeling on task data obtained from a sample of 11- and 12-year old children. Consistent with hypotheses, variance in both the Response Inhibition and Attentional Inhibition factors was significantly explained by individual differences in WMC. As further predicted, the Response Inhibition and Attentional Inhibition factors were empirically independent constructs after their shared statistical dependence on WMC was accounted for in the structural regression model. However, results obtained from the initial measurement model revealed that the correlation between the Response Inhibition and Attentional Inhibition factors was modest and only marginally significant. Furthermore, the empirical fit of a more constrained nested model demonstrated that the Response Inhibition and Attentional Inhibition factors could be represented as empirically independent constructs prior to being regressed onto the WMC factor. Stahl et al. (2014) previously found that their analogous constructs Stimulus Interference, measured using SS-conflict obtained on matching tasks, and Behavioral Inhibition, measured using non-selective stopping tasks, were not significantly correlated. The weak correlation found in the current study may similarly reflect success in using SS-conflict to capture unique variance in Attentional Inhibition, enabling it to be empirically differentiated from Response Inhibition. Given the low construct reliability of the Response Inhibition and Attentional Inhibition factors, their empirical independence may also reflect poor construct representation. The directional dependence between WMC and Attentional Inhibition factors could also not be conclusively determined, which may also reflect low construct reliability and low statistical power associated with small sample size.

We chose to focus on a narrow age range in the current study to reduce the impact of developmental changes on the results (Wiebe et al., 2008). The underlying neural networks supporting inhibitory control undergo increased functional segregation and independence during late childhood and early adolescence (Fair et al., 2007; Power et al., 2010; Luna et al., 2015). At a cognitive level, working memory and response inhibition exhibit divergent developmental trajectories, suggesting the observed relationships between the cognitive abilities in the proposed hierarchical model may change over the course of development (De Luca et al., 2003; Best and Miller, 2010; Lee et al., 2013). One possibility is that increased functional segregation of neural networks during development results in greater differentiation of individual inhibitory control mechanisms at the behavioral level. Evidence for this is suggested by findings that tasks related to executive control tend to coalesce as a single factor in young children, but form a multi-factor construct in adults (Miyake et al., 2000; Wiebe et al., 2008; Allan and Lonigan, 2011; Brydges et al., 2012). Thus, the preliminary results reported in this study require replication and comparison across different developmental periods.

Notwithstanding these limitations, the current study is the first to demonstrate that Response Inhibition and Attentional Inhibition are empirically independent cognitive constructs in a developmental sample. A lack of dissociation between these two constructs in previous studies may be attributable to the use of cognitive tasks with low reliability and specificity. Previous studies have tended to conceptualize and use variants of the Flanker task as a measure of Attentional Inhibition (Friedman and Miyake, 2004; Diamond, 2013; Kane et al., 2016). However, previous studies and current results suggest that the Flanker Compatibility Effect (i.e., interference effect) is predominantly attributable to SR-conflict, not SS-conflict (Eriksen and Eriksen, 1974; Eriksen, 1995; van Veen et al., 2001). Using Flanker tasks as a measure of SS-conflict is likely to reduce the amount of ability-specific variance that can be extracted to derive a latent variable for Attentional Inhibition. These findings may explain why previous studies have obtained only weak factor loadings for the Attentional Inhibition factor, which also makes it more difficult to dissociate empirically from other constructs (Fornell and Larcker, 1981; Friedman and Miyake, 2004; Hair et al., 2006; Kane et al., 2016).

To our knowledge, ours is the first study to test and empirically support a hierarchical model of inhibitory control. The structural organization of the proposed model is consistent with the unified model of inhibitory control proposed by Munakata et al. (2011), in which directed global inhibition (i.e., Response Inhibition) and indirect competitive inhibition (i.e., Attentional Inhibition) are proposed to be independent neurobiological mechanisms apart from a shared reliance on goal-maintenance in prefrontal cortex. The hierarchical model of inhibitory control is similarly consistent with proposed biased competition (Desimone and Duncan, 1995) and top-down excitatory biasing mechanisms (Miller and Cohen, 2001; Herd et al., 2006; Munakata et al., 2011) of cognitive control. According to these accounts, goal-relevant neural representations (e.g., motivational context, task rules, stimulus–response associations, object category, color, shape, or location) are maintained in regions of the prefrontal cortex implicated in working memory as an ‘attentional set’ or ‘attentional template,’ which lowers activation thresholds in posterior cortices. In turn, this may bias selective processing of task-relevant stimuli and context-appropriate responding (Desimone and Duncan, 1995; Corbetta and Shulman, 2002; Egner and Hirsch, 2005; Silk et al., 2010).

Specification of WMC as a higher-order factor supporting inhibitory control is also consistent with experimental research highlighting the dependence of response inhibition and visual selective attention on WMC (Roberts et al., 1994; Awh et al., 1998; Downing, 2000; Nieuwenhuis et al., 2004; Unsworth et al., 2004; Lavie, 2005; Dillon and Pizzagalli, 2007; Burnham et al., 2014; Vandierendonck, 2014). Response inhibition is dependent upon maintenance of the task goal in working memory, consisting of a representation of the context in which the automatically primed response should be inhibited (Dillon and Pizzagalli, 2007; Munakata et al., 2011). Failure to maintain the task goal at sufficient levels of activation is believed to result in failed inhibition and execution of the automatic response (Nieuwenhuis et al., 2004; Dillon and Pizzagalli, 2007). Empirical evidence supports the role of working memory in response inhibition; participants with lower working memory spans demonstrate poorer inhibitory control and increases in working memory load lead to decrements in response inhibition (Roberts et al., 1994; Kane et al., 2001; Unsworth et al., 2004). The proposed dependency of Attentional Inhibition on the maintenance of information in working memory is also supported by research demonstrating behavioral overlap between these two cognitive functions (Vandierendonck, 2014). Maintaining a stimulus or pre-cued location in working memory facilitates visual selective attention to target stimuli (Awh et al., 1998; Downing, 2000). Conversely, increasing working memory load, thereby allocating attentional resources away from goal maintenance, leads to increased interference from salient, but task-irrelevant distractors (Lavie, 2005; Burnham et al., 2014). In combination, these findings suggest that Response Inhibition and Attentional Inhibition share a common source of variance in relying on active goal-maintenance in working memory.

### Theoretical and Practical Implications

Working memory capacity corresponds with the Central Executive in the multi-component model of working memory, originally described by Baddeley and Hitch (1974; Repovs and Baddeley, 2006; Kane et al., 2007). The Central Executive is a domain-general, attentional resource that regulates the function of lower-order cognitive components, such as auditory-verbal and visuospatial short-term memory (Baddeley and Hitch, 1974; Baddeley, 1996, 2012). Baddeley (1996, 2002, 2012) further proposed that the Central Executive could be fractionated into several functional subcomponents, such as the ability to focus attention. The hierarchical model of inhibitory control is therefore theoretically consistent with initial accounts of the Central Executive as a domain-general resource that supports lower-order, domain-specific attentional processes (Baddeley, 1996, 2002, 2012). The findings of the current study suggest that a hierarchical organization, with WMC specified as a higher-order ability, may encompass additional inhibitory control abilities, such as ‘Resistance to Proactive Interference’ (Rosen and Engle, 1998; Kane and Engle, 2000).

Taxonomies of inhibitory control previously recognized a distinction between the ability to ignore irrelevant stimuli and the ability to withhold a prepotent motor response (Wilson and Kipp, 1998; Dempster and Corkill, 1999b; Nigg, 2000). Friedman and Miyake (2004) challenged this distinction and contended that the ability to resist interference from irrelevant information in the environment and the ability to inhibit a prepotent motor response could be considered a unitary ability. Our results support those of previous studies (Stahl et al., 2014; Kane et al., 2016) and recent taxonomies in suggesting the need to differentiate between these two putative inhibitory control functions (Lustig et al., 2007; Diamond, 2013; Nigg, 2017). The present findings also indicate that Attentional Inhibition is not isomorphic with working memory, reflecting a common construct of ‘focused attention’ as has been previously suggested (Diamond, 2013).

Theoretical and empirical distinctions between these two inhibitory control abilities and WMC may assist in providing conceptual clarity to studies of cognitive development and developmental psychopathology. For example, the developmental trajectory of Attentional Inhibition has not yet been investigated, because it has not been traditionally distinguished from WMC and Response Inhibition. Researchers have suggested that WMC and Attentional Inhibition are largely overlapping constructs, and that greater WMC may thus support more efficient visual selective attention (Stedron et al., 2005; Gazzaley and Nobre, 2012; Vandierendonck, 2014). The current study demonstrated that the non-executive component of Attentional Inhibition, which was not shared with WMC, represented a larger proportion of variance in this ability, introducing the possibility of functional modularity and divergent developmental improvements. Some researchers have claimed that age-related improvements in resistance to interference (i.e., Attentional Inhibition) are important to normal cognitive development and that individual differences in this ability may underlie variation in general intelligence (Bjorklund and Harnishfeger, 1990; Dempster, 1991, 1992; Dempster and Corkill, 1999a; Lustig et al., 2007). A unique role for deficits in Response Inhibition and Attentional Inhibition underlying self-regulatory failure and the pathogenesis of developmental psychopathology has also been suggested (Nigg, 2000, 2017). Clarification of the relationship between Attentional Inhibition, WMC, and Response Inhibition provides a foundation for these theories to be addressed in subsequent studies.

A potentially important practical implication of the hierarchical model of inhibitory control is in relation to the neuropsychological profile and clinical presentation of children with low WMC. The current model predicts that children with deficits in WMC may also exhibit concomitant difficulties in selectively attending to task-relevant stimuli and inhibiting behavioral responses. Such predictions are in keeping with the cognitive and behavioral profile of children with poor WMC, including those with Attention Deficit Hyperactivity Disorder (ADHD) (Barkley, 1997; Gathercole and Alloway, 2008; Alloway, 2010). Conversely, the small empirical overlap of Attentional Inhibition with WMC suggest that difficulties in ignoring distracting stimuli, such as those observed in educational contexts, may not be attributable to limits in WMC per se (Gathercole and Alloway, 2008; Alloway et al., 2009). Independent assessment of Attentional Inhibition as a distinct cognitive mechanism may reveal circumscribed deficits in attentional capacity with implications for classroom learning (Gathercole and Alloway, 2008; Alloway, 2010). Similarly, WMC predicted only half the variance in Response Inhibition in the hierarchical model of inhibitory control. The discriminant validity of these constructs suggests distinctions between attention, learning, and memory difficulties on one hand and problems of impulsivity and behavioral control on the other, with potential implications for more tailored educational assessment and intervention.

The hierarchical model of inhibitory control predicts that response inhibition is a distinct neurocognitive mechanism from active goal maintenance in working memory. This perspective challenges the prominent Unity/Diversity model of executive function in which variance associated with inhibiting prepotent behavioral responses is completely subsumed by a general goal maintenance factor (Friedman et al., 2008, 2011; Miyake and Friedman, 2012). Thus, according to the Unity/Diversity model, response inhibition is an epiphenomenon of goal maintenance processes rather than a unique cognitive ability (Miyake and Friedman, 2012; Snyder et al., 2015). This model also predicts that cognitive impairments observed across different forms of psychopathology are associated with relatively uniform deficits in goal maintenance (Snyder et al., 2015). In contrast, the current study demonstrated that goal maintenance in working memory (i.e., WMC) and response inhibition were related, but distinct cognitive constructs, suggesting the possibility of unique relationships with different forms of developmental psychopathology (Aron and Poldrack, 2005; Lipszyc and Schachar, 2010; Huang-Pollock et al., 2017).

From a research and clinical perspective, the current study indicates the need for independent assessment of Attentional Inhibition, Response Inhibition, and WMC, using multiple tasks where possible to surmount the task impurity problem (Friedman and Miyake, 2004; Snyder et al., 2015). We recommend that visual matching tasks or manual Stroop tasks, with minimal SR-conflict, be used to independently assess Attentional Inhibition (Kornblum et al., 1990; De Houwer, 2003; Stahl et al., 2014). Conversely, our results suggest that Flanker tasks may not measure Attentional Inhibition as previously contended (Friedman and Miyake, 2004; Diamond, 2013), and therefore we caution their use to asses this construct. WMC and Response Inhibition should also be assessed independently using complex memory span and non-selective stopping tasks, respectively. The statistical dependence of the Response Inhibition factor on WMC should be considered when evaluating the relationship between response inhibition and other variables. A related implication is the use of cognitive training for remediating symptoms of disinhibitory psychopathology. Impairments in response inhibition have been implicated as a potential neurocognitive endophenotype in ADHD (Aron and Poldrack, 2005; Robbins et al., 2012). However, cognitive interventions for ADHD in children and adolescents have often been targeted toward improving working memory (Klingberg et al., 2002, 2005; Klingberg, 2010). The current model predicts that working memory training would not fully translate to improvements in inhibitory control, because WMC shares only half of its variance with response inhibition. Interventions combining working memory and response inhibition training may therefore be more effective for remediating behavioral problems in disinhibitory psychopathology (Johnstone et al., 2012; Spierer et al., 2013).

### Limitations

Identification of the three cognitive constructs in the current study was based upon a synthesis of previous factor analytic studies, which have demonstrated patterns of intercorrelations in task performance largely consistent with the separate factors identified in the current model (Friedman and Miyake, 2004; Alloway et al., 2006; Kane et al., 2016). Nevertheless, studies combining a correlational and experimental methodology would be needed to establish the validity of the three identified constructs. Furthermore, the construct reliability of the two inhibitory control factors was not optimal and the Attentional Inhibition factor, in particular, was not well-identified. In part, this reflected the task impurity problem, as well as the fact that the Flanker task did not provide a good measure of the latent variable (Friedman and Miyake, 2004). Replication of the current findings using a greater number of observed variables and more robust indicators of the constructs of interest is required to draw firm conclusions regarding the utility of the proposed model. Although some studies have used multiple variants of the matching task, this may be problematic due to shared task-specific variance (Friedman and Miyake, 2004; Stahl et al., 2014; Kane et al., 2016). Thus, identifying an appropriate set of indicators to measure the Attentional Inhibition factor is indicative more broadly of a paucity of well-designed tasks for measuring complex cognitive processes (Friedman and Miyake, 2004).

Response Inhibition and Attentional Inhibition have both been categorized under the broader rubric of ‘*executive function*,’ a multidimensional construct encompassing several interrelated, higher-order processes involved in the goal-directed control of cognition and behavior (Diamond, 2013; Nigg, 2017). Several researchers have questioned the utility of executive functions constructs more generally, suggesting they lack discriminant validity and may reflect existing and well-established non-executive cognitive processes (Salthouse, 2005; van der Sluis et al., 2007; Martinez et al., 2011). A limitation of the current study was that validated measures of possibly related cognitive constructs, such as auditory-verbal short-term memory, were not included to ensure the inhibitory control constructs had discriminant validity (Colom et al., 2005; Martinez et al., 2011). Additionally, sample characteristics may restrict the generalizability of the present findings. The current sample size was comparable to that used by Miyake et al. (2000) (*N* = 137) in first establishing the Unity/Diversity model of executive function. However, the use of a larger sample is needed for sufficient power to compare competing nested models, validate the precision of the parameter estimates, as well as assess the adequacy of model fit to the broader population (MacCallum et al., 1996; Hermida et al., 2015; Kline, 2016). Use of a developmental sample may have introduced limitations to the generalizability of the model to other age groups due to the protracted development of the neural networks underlying working memory and response inhibition, as well as their divergent developmental trajectories (Klingberg, 2006; Best and Miller, 2010; Hwang et al., 2010; Luna et al., 2015). The hierarchical model of inhibitory control therefore requires replication in participant samples drawn from across a broader developmental spectrum.

## Conclusion

The current study introduced and tested a hierarchical model of inhibitory control. The results challenge existing models and theories of inhibitory control in two respects. First, it was demonstrated that Response Inhibition and Attentional Inhibition both exhibit a statistical dependence on WMC, consistent with cognitive and neurobiological models of top-down control. Second, the Response Inhibition and Attentional Inhibition factors could be modeled as empirically independent constructs, suggesting that they are distinct, and perhaps functionally unrelated, cognitive abilities. The hierarchical model of inhibitory control may provide a useful conceptual framework for future theorizing and empirical studies examining the relationship between these processes and other aspects of cognitive, affective, and behavioral functioning, as well as how they change across the course of development.

## Data Availability

The dataset analyzed for this study can be found in PsyArXiv Open Science Framework [https://osf.io/u2w79/].

## Author Contributions

JT was responsible for conceptualizing and designing the study, data collection, analyses, and write-up. RT, MB, CP, and SW advised on study design, data collection, interpretation of results, and write-up of the manuscript. All authors contributed to manuscript revision, as well as read and approved the final manuscript.

## Conflict of Interest Statement

The authors declare that the research was conducted in the absence of any commercial or financial relationships that could be construed as a potential conflict of interest.
